# Synergistic impact of nutritional risk, glycemic control, and systemic inflammation on Abdominal Compartment Syndrome in diabetic patients following complex ventral hernia repair: a development and validation study

**DOI:** 10.3389/fnut.2026.1786526

**Published:** 2026-06-19

**Authors:** Xue Zhang, Yingmo Shen, Baoshan Wang, Jie Chen

**Affiliations:** 1 Department of Dermatology and Aesthetic Medicine, Beijing Chao-Yang Hospital, Capital Medical University, Beijing, China; 2 Department of Hernia and Abdominal Surgery, Beijing Chao-Yang Hospital, Capital Medical University, Beijing, China

**Keywords:** Abdominal Compartment Syndrome, clinical prediction model, HbA1c, immuno-nutrition, NRS-2002, SIRI

## Abstract

**Background:**

Abdominal Compartment Syndrome (ACS) is a catastrophic complication following complex hernia repair, particularly in patients with Type 2 Diabetes Mellitus (T2DM) who exhibit a phenotype of “metabolic vulnerability.” The interplay between preoperative nutritional depletion, chronic hyperglycemia, and systemic inflammation remains poorly understood in this context. Current risk assessment tools rely heavily on anatomical metrics and often fail to capture the synergistic impact of immuno-metabolic fragility. We aimed to develop and prospectively temporally validate a dynamic nomogram that integrates immuno-nutritional markers with surgical variables to predict ACS in diabetic patients.

**Methods:**

We conducted a two-stage, prospective temporal validation study involving 555 diabetic patients undergoing elective complex hernia repair at a tertiary referral center. Phase I (January 2015 to December 2021) comprised a retrospective derivation cohort (*N* = 461) that was randomly split into a training set (*n* = 323) and an internal testing set (*n* = 138) to identify predictors and construct the model. Phase II (January 2022 to December 2024) established a prospective temporal validation cohort (*N* = 94) to verify model performance in a real-world clinical setting at the same institution. We utilized determining factors including the Hernia Sac Volume to Abdominal Cavity Volume (HSV/ACV) ratio, Nutritional Risk Screening 2002 (NRS-2002), Systemic Inflammatory Response Index (SIRI), and Glycated Hemoglobin (HbA1c). The primary endpoint was the development of ACS within 7 days postoperatively. Pre-specified sensitivity analyses included LASSO penalized regression with bootstrap optimism correction (1,000 resamples), modeling of all continuous predictors as restricted cubic splines, and comparison of nested models (anatomical-only vs. anatomical + metabolic vs. full nomogram) by decision curve analysis, net reclassification improvement (NRI), and integrated discrimination improvement (IDI).

**Results:**

The study population exhibited a high baseline metabolic burden, with a mean body-mass index of 31.2 kg/m^2^ and a mean HbA1c of 7.9%. Multivariable logistic regression identified seven independent predictors: HSV/ACV ratio ≥ 0.25 (Odds Ratio [OR], 2.75; 95% Confidence Interval [CI], 1.60–4.85), use of tension reduction procedures (OR, 2.45), operative time > 200 min (OR, 2.12), BMI ≥ 30 kg/m^2^ (OR, 1.88), NRS-2002 score ≥ 3 (OR, 2.18), SIRI ≥ 1.6 (OR, 1.98), and HbA1c ≥ 6.0% (OR, 1.65). A positive correlation between SIRI and nutritional risk (Spearman ρ = 0.42, *P* < 0.001) was observed, consistent with an immuno-nutritional axis. The nomogram demonstrated good and stable discrimination, with an Area Under the Curve (AUC) of 0.89 (95% CI, 0.85–0.93) in the training cohort, 0.86 in the internal testing cohort, and 0.84 (95% CI, 0.77–0.91) in the prospective temporal validation cohort. Comprehensive calibration assessment in the prospective cohort showed a calibration slope of 0.91 (95% CI 0.74–1.08), calibration intercept of −0.09 (−0.31 to 0.13), Brier score 0.112, and a non-significant Hosmer–Lemeshow test (*P* = 0.45). Bootstrap-based internal validation (1,000 resamples) yielded an optimism-corrected C-index of 0.87 and a calibration slope of 0.93. Risk stratification categorized patients into low, intermediate, and high-risk groups, yielding ACS incidence rates of 1.0%, 10.1%, and 72.7%, respectively (*P* < 0.001). Compared with the anatomical-only baseline model, the full nomogram achieved an NRI of 0.31 (95% CI 0.18–0.44) and an IDI of 0.094 (95% CI 0.061–0.127), both *P* < 0.001. The high-risk phenotype was associated with significantly greater postoperative fluid sequestration, prolonged intensive care unit stays, and increased 30-days mortality.

**Conclusion:**

The integration of immuno-nutritional markers (SIRI, NRS-2002) and glycemic control with anatomical parameters provides a temporally validated tool with good and stable discrimination for ACS in diabetic patients. Its principal clinical utility lies in reliable risk exclusion (negative predictive value 95.5% in the prospective temporal validation cohort), supporting safe rule-out of low-risk patients, while the more modest positive predictive value (46.4%) indicates that a high score should prompt enhanced surveillance and individualized decision-making rather than constitute a deterministic indication for pre-emptive open-abdomen management.

## 1 Introduction

Complex abdominal wall reconstruction (AWR) represents one of the most technically challenging frontiers in modern surgery, particularly for patients with massive incisional hernias and loss of domain (LOD). While advancements in component separation techniques (CST) and mesh biotechnology have improved anatomical closure rates, the physiological consequences of returning large visceral volumes into a stiff, non-compliant abdomen remain perilous (1, 2). The most devastating of these sequelae is Abdominal Compartment Syndrome (ACS), a state of sustained intra-abdominal hypertension (IAH) that triggers multiorgan failure, characterized by a precipitous mortality rate exceeding 40% even with aggressive decompression (3, 4). The challenge is exponentially amplified in the rapidly expanding demographic of patients with Type 2 Diabetes Mellitus (T2DM). Diabetic patients are not merely individuals with hyperglycemia; they represent a distinct phenotype of “metabolic vulnerability,” characterized by microvascular fragility, impaired tissue perfusion, and a chronic, low-grade inflammatory state (5). Despite this, current risk stratification models for ACS remain dominated by what we term a structural-volumetric paradigm, defined as a framework that quantifies perioperative risk almost exclusively through static morphometric parameters of the abdominal wall and herniated viscera–such as fascial defect width, hernia sac dimensions, and the Hernia Sac Volume to Abdominal Cavity Volume (HSV/ACV) ratio–while disregarding the dynamic immuno-metabolic reserve of the host. This paradigm has been epitomized by the Tanaka index and its derivatives (6, 7). While the “Tanaka index” and its derivatives effectively quantify the mechanical mismatch, they fundamentally fail to account for the host’s biological resilience–specifically, the physiological capacity of the diabetic patient to withstand the massive hemodynamic and respiratory shifts induced by fascial closure (8). As the prevalence of diabesity (diabetes combined with obesity) surges globally, there is an urgent imperative to move beyond purely anatomical prediction rules toward a multidimensional paradigm that integrates biomechanics with metabolic competence.

The pathophysiology of ACS in the diabetic surgical patient is hypothesized to involve a confluence of immuno-metabolic dysregulation, which we frame conceptually as the “Malnutrition-Inflammation Complex Syndrome” (MICS) in the context of AWR (9). This proposed mechanism extends beyond simple hydrostatic pressure. Chronic hyperglycemia and insulin resistance are reported to compromise the endothelial glycocalyx, increasing systemic capillary permeability and predisposing tissues to distinct “third-spacing” fluid sequestration (10, 11). In this precarious environment, traditional markers like C-reactive protein (CRP) are often too non-specific. Conversely, novel composite biomarkers such as the Systemic Inflammatory Response Index (SIRI)–which integrates neutrophils, monocytes, and lymphocytes–offer a granular window into the balance between aggressive inflammation and immune regulation (12, 13). Elevated SIRI has been proposed to reflect a neutrophil-driven innate immune overactivation that exacerbates tissue edema, while simultaneous lymphopenia indicates physiological exhaustion. Crucially, this inflammatory burden is closely linked to nutritional status. Patients with unrecognized malnutrition (detectable via NRS-2002) lack the protein substrates, such as albumin, necessary to maintain oncotic pressure, which has been associated with visceral edema and intra-abdominal hypertension (14). Therefore, we hypothesize that ACS is not solely a mechanical consequence of “tight closure,” but may also reflect a systemic failure of the immuno-nutritional axis to compensate for surgical stress. Whether integrating these dynamic biological markers (SIRI, HbA1c, NRS-2002) with static anatomical constraints (HSV/ACV) can unmask the high-risk phenotype that anatomical volumetry alone overlooks remains to be established empirically.

Building upon our group’s previous investigations into abdominal wall biomechanics and tension-free repair strategies, this study aims to bridge the gap between anatomical volumetry and systemic physiology. We hypothesize that a “Metabolic-Anatomical” nomogram can predict the onset of ACS with superior accuracy compared to traditional models. To test this, we designed a rigorous two-stage study comprising a retrospective derivation cohort to identify candidate predictors, followed by a prospective temporal validation cohort to verify the model’s stability in real-world clinical flows. By quantifying the association between the HSV/ACV ratio and the immuno-nutritional triad of SIRI, NRS-2002, and HbA1c, we sought to develop a precision medicine tool capable of stratifying risk.

## 2 Study design and methods

### 2.1 Study design and participant recruitment

To ensure robust model development and rigorous validation, this study employed a two-stage, prospective temporal validation design, focusing specifically on a metabolically vulnerable cohort of patients with Type 2 Diabetes Mellitus (T2DM). The study protocol adhered to the STROBE guidelines for observational studies (15) and the TRIPOD statement for multivariable prediction models (16).

#### 2.1.1 Phase I: retrospective model derivation and internal testing (2015–2021)

The initial phase involved a retrospective analysis of prospectively maintained clinical databases at Beijing Chao-yang Hospital. A total of 461 eligible diabetic patients who underwent complex hernia repair between January 2015 and December 2021 were enrolled. To facilitate model development and internal validation, this primary cohort was randomly partitioned into two datasets using a computer-generated random number sequence (ratio of 7:3): (1) Training Cohort (*n* = 323): Used to identify independent immuno-nutritional predictors and construct the nomogram. (2) Internal Testing Cohort (*n* = 138): Used to evaluate the model’s reproducibility and prevent overfitting within the same temporal timeframe.

#### 2.1.2 Phase II: prospective temporal validation (2022–2024)

To verify the temporal stability of the model in a real-world clinical setting at the same institution, a prospective temporal validation cohort was established. From January 2022 to December 2024, we consecutively enrolled 94 diabetic patients adhering to the same inclusion criteria. In this prospective phase, nutritional screening (NRS-2002) and metabolic assessments were standardized and performed in real-time upon admission.

### 2.2 Inclusion and exclusion criteria

Eligible patients must meet the following criteria: (1) Confirmed Diagnosis of T2DM: Defined according to the World Health Organization (WHO) 1999 criteria: fasting plasma glucose ≥ 7.0 mmol/L (126 mg/dL), or 2-h plasma glucose ≥ 11.1 mmol/L (200 mg/dL) during an oral glucose tolerance test, or a prior diagnosis treated with antihyperglycemic medication or insulin. (2) Surgical Intervention: Patients aged ≥ 18 years undergoing elective open complex hernia repair (including incisional, ventral, or recurrent hernias requiring component separation or myofascial release). (3) Nutritional and Inflammatory Data Availability: Complete documentation of preoperative nutritional status (NRS-2002 score), inflammatory markers (neutrophil, lymphocyte, monocyte counts for SIRI/NLR), and glycemic control (HbA1c).

**FIGURE 1:**
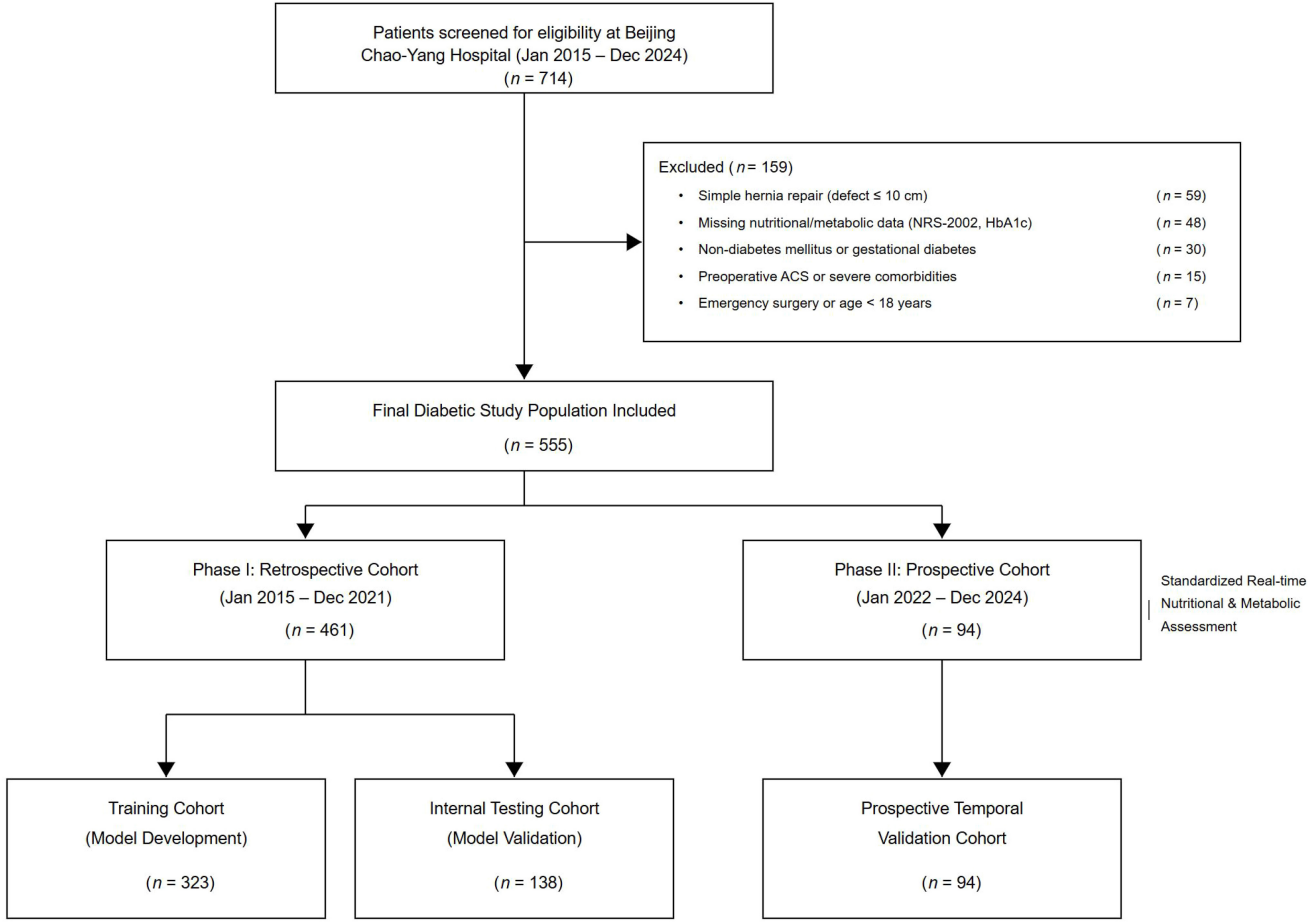
Study flowchart. Of 714 diabetic patients screened for eligibility at Beijing Chao-Yang Hospital between January 2015 and December 2024, 159 were excluded for the reasons itemized in the right-hand box (simple hernia repair with defect ≤ 10 cm, *n* = 59; missing nutritional or metabolic data including NRS-2002 or HbA1c, *n* = 48; non-Type 2 diabetes mellitus, *n* = 22; gestational diabetes, *n* = 8; preoperative ACS or severe comorbidities including Child–Pugh C cirrhosis [*n* = 3], end-stage renal disease on dialysis [*n* = 4], NYHA class IV heart failure [*n* = 2], and preoperative ACS [*n* = 6]; emergency surgery, *n* = 5; age < 18 years, *n* = 2), leaving 555 diabetic patients in the final study population. Phase I (retrospective derivation; January 2015–December 2021; *n* = 461) was randomly partitioned 7:3 into a training cohort (*n* = 323) for model development and an internal testing cohort (*n* = 138) for reproducibility assessment. Phase II established a prospective temporal validation cohort (January 2022–December 2024; *n* = 94) with standardized real-time nutritional and metabolic assessment. All patients were enrolled at a single tertiary referral center. The primary outcome was abdominal compartment syndrome (ACS) within 7 days postoperatively, defined as sustained intra-abdominal pressure > 20 mmHg with new-onset organ dysfunction; the total ACS incidence was 16.2% (*n* = 90), with comparable rates between Phase I (16.1%) and Phase II (17.0%). ACS, Abdominal Compartment Syndrome; ESRD, end-stage renal disease; HbA1c, glycated hemoglobin; NRS-2002, Nutritional Risk Screening 2002; NYHA, New York Heart Association; T2DM, type 2 diabetes mellitus.

Patients were excluded if they presented with confounding factors that could independently alter inflammatory or nutritional markers, including: (1) Emergency surgery or trauma (acute stress state). (2) Active malignancy or chemotherapy/radiotherapy within the past 6 months (catabolic state). (3) Chronic steroid use or immunosuppressive therapy (interference with inflammatory markers like SIRI). (4) Incomplete data regarding key metabolic or nutritional indicators (e.g., missing HbA1c, albumin, or differential blood counts). The granular breakdown of all exclusions (*n* = 159) is detailed in Figure 1, with 59 patients excluded for simple hernia repair (defect ≤ 10 cm), 48 for missing nutritional/metabolic data, 30 for non-T2DM/gestational diabetes, 15 for preoperative ACS or severe comorbidities (Child–Pugh C cirrhosis, end-stage renal disease on dialysis, NYHA class IV heart failure), 5 for emergency surgery, and 2 for age < 18 years.

The study protocol was reviewed and approved by the Institutional Review Board (IRB) (Approval No.: 2021-K-656). For Phase I (Retrospective), the requirement for informed consent was waived. For Phase II (Prospective), written informed consent was obtained from all participants prior to data collection (Figure 1).

### 2.3 Data collection

Data were extracted from electronic medical records, surgical registries, and laboratory reports. A standardized case report form was used to record demographic data [age, sex, body mass index (BMI), chronic obstructive pulmonary disease (COPD)], diabetes-related variables (HbA1c, duration of diabetes, insulin use), hernia characteristics [defect size, area loss, recurrence status, Hernia Sac Volume relative to Abdominal Cavity Volume (HSV/ACV), IAP], surgical details [operative duration, tension reduction procedure (TRP), estimated blood loss, intraoperative complications], nutritional indicators [serum albumin, Nutritional Risk Screening 2002 (NRS-2002) score], inflammatory markers [neutrophil-to-lymphocyte ratio (NLR), systemic inflammatory response index (SIRI), C-reactive protein (CRP)], and biochemical parameters (lactate, white blood cell count). NLR and SIRI were calculated based on the preoperative complete blood count.

### 2.4 Assessment of nutritional and inflammatory profiles

To comprehensively evaluate the biological reserve and stress resilience of this diabetic cohort, a multidimensional assessment involving nutritional screening and immuno-nutritional profiling was performed within 24 h of hospital admission.

#### 2.4.1 Nutritional Risk Screening (NRS-2002)

Baseline nutritional risk was quantified using the Nutritional Risk Screening 2002 (NRS-2002) tool, a validated instrument recommended by the European Society for Clinical Nutrition and Metabolism (ESPEN). This composite score (range 0–7) evaluates the patient’s capacity to withstand surgical stress by summing three components: (1) Impaired Nutritional Status (0–3 points): Based on the severity of weight loss (>5% in 3 months), reduced dietary intake (0%–100% of requirements), and low BMI. (2) Disease Severity (0–3 points): Reflecting the catabolic stress induced by the underlying pathology (e.g., major abdominal surgery confers 2 points). (3) Age Adjustment: An additional 1 point is added for patients aged ≥ 70 years. A total score of ≥3 was defined as “nutritional risk,” indicating a compromised physiological reserve susceptible to adverse outcomes.

#### 2.4.2 Immuno-nutritional markers (SIRI)

Systemic inflammation and immune competence were assessed using the Systemic Inflammatory Response Index (SIRI), a novel composite biomarker reflecting the balance between the host’s inflammatory drive and nutritional-immune status. SIRI was calculated using absolute peripheral blood cell counts derived from the first fasting blood sample upon admission, defined as: SIRI = (Neutrophil count × Monocyte count)/Lymphocyte count.

### 2.5 Volumetric assessment by CT

**FIGURE 2:**
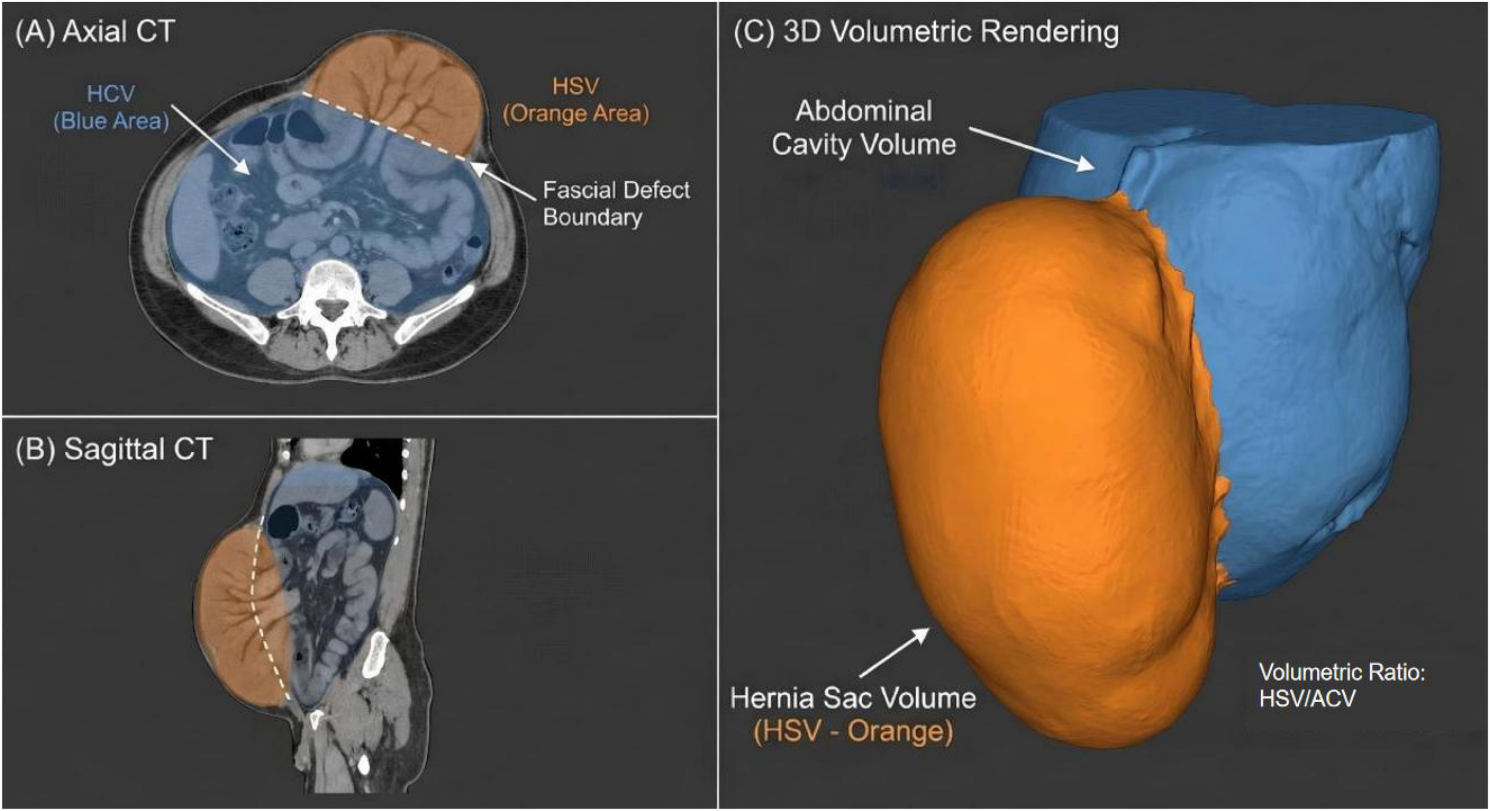
Volumetric assessment of Hernia Sac Volume (HSV) and Abdominal Cavity Volume (ACV) using the Tanaka method. **(A)** Axial contrast-enhanced computed tomography image demonstrating the segmentation method. The Hernia Cavity Volume (ACV, blue) is defined as the space delimited superiorly by the diaphragm, inferiorly by the pelvic floor, and circumferentially by the proper abdominal wall fascia. The Hernia Sac Volume (HSV, orange) represents the volume of eventrated viscera protruding beyond the fascial defect line. The dashed red line indicates the fascial defect boundary. Anatomical orientation indicators (R, right; L, left; A, anterior; P, posterior) are shown. Scale bar = 5 cm. **(B)** Sagittal reconstruction providing a longitudinal view of the hernia defect and visceral content distribution; cranial, caudal, and dorsal directions are annotated. Scale bar = 5 cm. **(C)** Three-dimensional volumetric rendering used to compute the HSV/ACV ratio; severe loss of domain (LOD) was defined as HSV/ACV ratio ≥ 0.25 per Tanaka et al. ICC, intra-class correlation coefficient; LoA, limits of agreement; LOD, loss of domain.

Volumetric assessment was conducted using preoperative contrast-enhanced computed tomography (CT) scans, based on the established method described by Tanaka et al. Utilizing semi-automated segmentation software, the Abdominal Cavity Volume (ACV), delimited by the boundaries of the proper abdominal wall fascia, the diaphragm superiorly, and the pelvic floor inferiorly, and the Hernia Sac Volume (HSV), representing the entirety of the eventrated viscera protruding beyond the fascial defect plane, were quantified independently. The HSV/ACV ratio was subsequently calculated to determine the extent of visceral protrusion; adhering to Tanaka’s classical definition, a ratio exceeding 25% was used as the threshold to identify significant Loss of Domain (LOD). The HSV/ACV ratio was subsequently calculated to determine the extent of visceral protrusion; adhering to Tanaka’s classical definition, a ratio exceeding 25% was utilized as the threshold to identify significant “Loss of Domain (LOD)” (Figure 2). Volumetric segmentation was performed independently by two board-certified radiologists blinded to clinical outcomes. Inter-observer reproducibility, assessed in a random 10% subsample (*n* = 56), yielded an intra-class correlation coefficient (ICC, two-way random effects, single rater, absolute agreement) of 0.94 (95% CI 0.91–0.96) for ACV, 0.92 (0.88–0.95) for HSV, and 0.93 (0.90–0.95) for the HSV/ACV ratio. Bland–Altman analysis showed a mean bias of −1.2% with 95% limits of agreement of −5.8% to +3.4% for the HSV/ACV ratio.

### 2.6 Outcome definition: metabolic decompensation and compartment syndrome

The primary endpoint was ACS, defined as sustained intra-abdominal pressure (IAP) > 20 mmHg with new-onset organ dysfunction (respiratory, renal, or cardiovascular failure) within 7 days of surgery according to the World Society of Abdominal Compartment Syndrome criteria. Secondary outcomes were length of ICU stay, 30-days mortality, ventilator support requirement, surgical site infection (SSI), postoperative hyperglycemia, net 24-h fluid balance, and 30-days readmission.

### 2.7 Statistical analysis

#### 2.7.1 Descriptive statistics and group comparisons

Continuous variables were first assessed for normality using the Shapiro-Wilk test and visual inspection of histograms. Normally distributed data were expressed as means ± standard deviations (SD), while non-normally distributed data were presented as medians with interquartile ranges (IQR). Categorical variables were summarized as frequencies and percentages. Baseline characteristics and postoperative outcomes were compared between groups (Training vs. Testing; ACS vs. Non-ACS; Risk Strata) using the Student’s *t*-test or One-way ANOVA for normally distributed continuous variables, the Mann-Whitney U test or Kruskal-Wallis H test for non-normally distributed variables, and the Chi-square test (χ^2^) or Fisher’s exact test for categorical variables, as appropriate. The standardized mean difference (SMD) between the pooled Phase I derivation cohort and the Phase II prospective temporal validation cohort was computed for all baseline variables to quantify between-cohort balance, with SMD < 0.10 interpreted as negligible imbalance and SMD < 0.20 considered acceptable for transportability.

#### 2.7.2 Correlation analysis of immuno-nutritional markers

To investigate the “Malnutrition-Inflammation Complex Syndrome” (MICS) in this population, a Spearman’s rank correlation analysis was conducted. A correlation heatmap was generated to visualize the pairwise relationships between key nutritional (NRS-2002, Albumin), inflammatory (SIRI, NLR), and metabolic (HbA1c, BMI) indicators. As correlation analyses are not informative about causal directionality, this analysis was framed as descriptive and hypothesis-generating, with mechanistic and causal pathways considered to require confirmation in dedicated translational and interventional studies (see Section “4 Discussion”).

#### 2.7.3 Model development and variable selection

A multivariable logistic regression model was developed using the training cohort to predict the risk of ACS. The variable selection process followed a structured approach: (1) Univariate Screening: Potential predictors were initially screened using univariate logistic regression; variables with a *P*-value < 0.10 were considered candidates for the multivariable model. (2) Linearity Assessment: The linearity assumption for continuous variables (HSV/ACV ratio, BMI, operative time, SIRI, HbA1c) was evaluated using restricted cubic splines (RCS, 3 knots placed at the 10th, 50th, and 90th percentiles) with likelihood-ratio tests against the linear specification. Predictors with significant non-linearity (*P* < 0.05) or RCS inflection points consistent with established clinical thresholds were dichotomized at biologically and clinically meaningful cut-offs (HSV/ACV ratio ≥ 0.25 [Tanaka], BMI ≥ 30 kg/m^2^ [WHO], operative time > 200 min [Youden], SIRI ≥ 1.6 [Youden + RCS inflection], HbA1c ≥ 6.0% [American Diabetes Association]). The choice of these cut-offs is justified in detail in Supplementary Figure 1 and Supplementary Table 5. We acknowledge that dichotomization reduces statistical efficiency; therefore, a sensitivity analysis treating BMI, operative time, SIRI, and HbA1c as continuous variables was performed (Supplementary Table 4). (3) Multivariable Modeling: A backward stepwise selection procedure based on the Akaike Information Criterion (AIC) was employed to identify independent predictors. (4) Collinearity Check: Multicollinearity among predictors was assessed using the Variance Inflation Factor (VIF), with a threshold of VIF < 5 indicating no significant collinearity (Supplementary Table 3). (5) Nomogram Construction: A prognostic nomogram was constructed based on the regression coefficients of the final independent predictors.

#### 2.7.4 Sensitivity analyses and optimism correction

To address potential overfitting and optimism bias, three pre-specified sensitivity analyses were performed. First, the events-per-variable (EPV) ratio was calculated for the training cohort and the full Phase I derivation cohort (Supplementary Table 1). Second, LASSO penalized logistic regression with 10-fold cross-validation (1-SE rule) was used to re-estimate the regression coefficients with shrinkage; the variables retained and the resulting coefficients were compared with the AIC-selected model (Supplementary Table 2). Third, internal validation by 1,000 bootstrap resamples was performed using the validate function in the rms R package to estimate the optimism in the apparent C-index, the Van Houwelingen calibration slope, and a uniform shrinkage factor that was used to recalibrate the model intercept (Supplementary Figure 3). Additionally, sensitivity analyses with alternative HbA1c cut-offs (6.0%, 6.5%, 7.0%) and with HbA1c as a continuous predictor were performed to assess threshold robustness (Supplementary Table 5).

#### 2.7.5 Model validation and performance metrics

**FIGURE 3:**
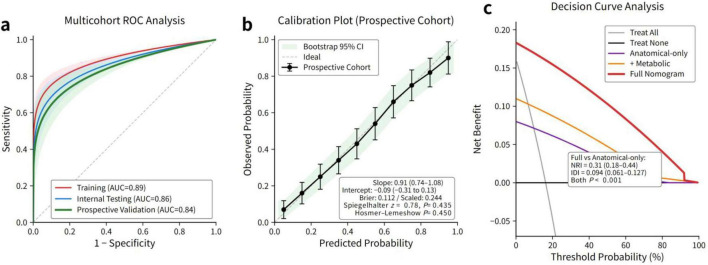
Multidimensional validation of the immuno-nutritional nomogram. **(a)** Receiver operating characteristic (ROC) curves with 95% confidence bands across study cohorts: training (red, AUC = 0.89; 95% CI 0.85–0.93), internal testing (blue, AUC = 0.86; 95% CI 0.80–0.92), and prospective temporal validation (green, AUC = 0.84; 95% CI 0.77–0.91). The 95% confidence bands are derived from DeLong’s variance estimator. Pairwise comparisons of AUC by DeLong’s test confirmed no significant deterioration between the training cohort and either of the validation cohorts (both *P* > 0.05), demonstrating good and stable discrimination across temporal cohorts. **(b)** Calibration plot for the prospective temporal validation cohort showing predicted versus observed probabilities by deciles of predicted risk, with bootstrap-derived 95% confidence band (light green shading) and per-bin error bars. The calibration slope (0.91; 95% CI 0.74–1.08), calibration intercept (–0.09; 95% CI –0.31 to 0.13), Brier score (0.112), and Hosmer–Lemeshow *P*-value (0.45) are annotated in the upper-left inset. The plotted line closely follows the diagonal reference (gray dashed) across the full range of predicted risk, with mild overestimation only in the highest decile (predicted probability ≥ 0.80), where the absolute number of patients was small. **(c)** Decision curve analysis showing net benefit across the threshold probability range for versus the treat-all and treat-none default strategies. Model 1 (anatomical-only, purple): HSV/ACV ratio + tension reduction procedure + operative time. Model 2 (anatomical + metabolic, teal): Model 1 + BMI + HbA1c. Model 3 (full nomogram, red): Model 2 + SIRI + NRS-2002. The full nomogram provide three nested models the highest net benefit across the clinically relevant threshold range (10%–60%). Net reclassification improvement (NRI) and integrated discrimination improvement (IDI) of the full nomogram versus the anatomical-only baseline are annotated at the bottom of the panel: NRI = 0.31 (95% CI 0.18–0.44) and IDI = 0.094 (95% CI 0.061–0.127), both *P* < 0.001. AUC, area under the curve; BMI, body mass index; CI, confidence interval; DCA, decision curve analysis; HbA1c, glycated hemoglobin; ACV, Abdominal Cavity Volume; HSV, Hernia Sac Volume; IDI, integrated discrimination improvement; NB, net benefit; NRI, net reclassification improvement; NRS-2002, Nutritional Risk Screening 2002; ROC, receiver operating characteristic; SIRI, systemic inflammatory response index; TRP, tension reduction procedure.

The performance of the prediction model was evaluated in the training, internal testing, and prospective temporal validation cohorts using four dimensions: (1) Discrimination: The area under the receiver operating characteristic curve (AUC-ROC) was calculated to quantify the model’s discriminatory ability. The DeLong test was used to compare the AUC-ROC values between cohorts to assess stability. Sensitivity, specificity, positive predictive value (PPV), and negative predictive value (NPV) were calculated at the optimal cut-off determined by the Youden Index. 95% confidence bands around the ROC curves were derived using DeLong-derived variance (Figure 3a). (2) Calibration: Calibration was assessed using four complementary metrics: (i) the calibration slope (ideal = 1.00) and calibration intercept (ideal = 0.00) from a logistic recalibration of the linear predictor; (ii) the Brier score and the scaled Brier score (Brier skill score), where Scaled Brier = 1 − (Brier_model/Brier_null) and Brier_null = π(1 −π) with π denoting the cohort-specific ACS prevalence; (iii) Spiegelhalter’s *z*-test of the difference between predicted and observed probabilities; and (iv) the Hosmer–Lemeshow goodness-of-fit test. Calibration plots with bootstrapped 95% confidence bands were generated for all three cohorts. Bootstrap-corrected calibration slope was estimated by 1,000 resamples in the training cohort. (3) Clinical Utility: Decision Curve Analysis (DCA) was performed for three nested models: Model 1 (anatomical-only: HSV/ACV ratio, tension reduction procedure, operative time); Model 2 (anatomical + metabolic: Model 1 plus BMI and HbA1c); Model 3 (full nomogram: Model 2 plus SIRI and NRS-2002). Net benefit was compared with the default “treat all” and “treat none” strategies across threshold probabilities of 1%–99%. (4) Reclassification: To quantify the incremental discrimination achieved by adding the immuno-nutritional axis, the net reclassification improvement (NRI) and integrated discrimination improvement (IDI) of the full nomogram versus the anatomical-only baseline model were calculated using the PredictABEL R package, with 95% confidence intervals derived by 1,000 bootstrap resamples.

#### 2.7.6 Risk stratification strategy

To facilitate clinical decision-making, a risk stratification system was established. The X-tile software (Version 3.6.1, Yale University) was utilized to determine the optimal cut-off values for the nomogram’s total risk score. Based on these cut-offs, the study population was categorized into three distinct prognostic groups: Low Risk, Intermediate Risk, and High Risk. Differences in ACS incidence and secondary outcomes across these strata were analyzed to validate the stratification’s clinical relevance. Missing data (<20%) were handled using multiple imputation. The primary R packages used included rms for regression and nomogram construction, pROC for ROC analysis, ResourceSelection for Hosmer-Lemeshow tests, dca.r for decision curve analysis, glmnet for LASSO penalized regression, PredictABEL for reclassification statistics, and mice for imputation. A two-sided *P*-value < 0.05 was considered statistically significant.

## 3 Results

### 3.1 Baseline characteristics and prognosis

As summarized in Table 1, the demographic, surgical, and metabolic characteristics were statistically balanced between the training and internal testing cohorts (*P* > 0.05 for all comparisons), confirming the homogeneity of the randomized splitting. Crucially, the study population exhibited a distinct phenotype of “metabolic vulnerability,” characterized by a high prevalence of Class I obesity (mean BMI > 31 kg/m^2^), suboptimal glycemic control (mean HbA1c 7.9%), and significant nutritional risk (31.2%–33.0% with NRS-2002 ≥ 3), underscoring the coexistence of metabolic overload and physiological depletion in these patients. Furthermore, the Phase II prospective temporal validation cohort (*n* = 94) demonstrated baseline profiles and ACS incidence rates (17.0%) that were highly consistent with the retrospective phase (16.1%), thereby validating its suitability as a robust prospective temporal validation testing set. Standardized mean differences (SMDs) between the pooled Phase I and Phase II cohorts were <0.15 for all variables, indicating excellent baseline balance between the derivation and the prospective temporal validation cohorts; the only variables with SMD ≥ 0.10 were the HSV/ACV ratio (SMD = 0.11) and SIRI (SMD = 0.13), both well below the 0.20 threshold conventionally considered to indicate non-trivial imbalance (Table 1).

**TABLE 1:** Baseline characteristics of the training, internal testing, and prospective temporal validation cohorts.

Variable	Training (*n* = 323)	Internal testing (*n* = 138)	Prospective temporal validation (*n* = 94)	*P*-value^a^	SMD^b^
Demographics					
Age, years (mean ± SD)	58.6 ± 11.9	57.9 ± 12.5	60.1 ± 11.4	0.562	0.13
Male sex, *n* (%)	166 (51.4)	69 (50.0)	49 (52.1)	0.789	0.04
Smoking history, *n* (%)	101 (31.3)	41 (29.7)	27 (28.7)	0.741	0.05
Metabolic and nutritional profiles					
BMI, kg/m^2^ (mean ± SD)	31.3 ± 4.9	31.0 ± 4.6	31.5 ± 5.1	0.534	0.04
Obesity (BMI ≥ 30 kg/m^2^), *n* (%)	175 (54.2)	73 (52.9)	52 (55.3)	0.798	0.03
HbA1c, % (mean ± SD)	7.9 ± 1.6	7.8 ± 1.4	8.0 ± 1.7	0.501	0.06
HbA1c ≥ 6.0%, *n* (%)	133 (41.2)	56 (40.6)	40 (42.6)	0.908	0.03
NRS-2002 ≥ 3, *n* (%)	102 (31.6)	42 (30.4)	31 (33.0)	0.795	0.03
Serum albumin, g/L (mean ± SD)	36.3 ± 5.2	36.6 ± 4.9	35.9 ± 5.4	0.558	0.09
SIRI, median [IQR]	1.95 [1.10–3.50]	1.88 [1.00–3.30]	2.01 [1.20–3.60]	0.345	0.13^c^
NLR, median [IQR]	2.88 [2.00–4.30]	2.79 [1.80–4.10]	2.92 [2.10–4.50]	0.412	0.05
Surgical factors					
Hernia defect width, cm (mean ± SD)	14.6 ± 4.3	14.3 ± 4.0	14.8 ± 4.5	0.476	0.07
HSV/ACV ratio, median [IQR]	0.24 [0.15–0.37]	0.22 [0.13–0.35]	0.25 [0.16–0.38]	0.289	0.11^c^
HSV/ACV ratio ≥ 0.25, *n* (%)	152 (47.1)	62 (44.9)	46 (48.9)	0.812	0.04
Operative time, min (mean ± SD)	218 ± 65	209 ± 58	221 ± 68	0.145	0.05
Operative time > 200 min, *n* (%)	168 (52.0)	67 (48.6)	51 (54.3)	0.687	0.06
Tension reduction procedure, *n* (%)	129 (39.9)	53 (38.4)	40 (42.6)	0.765	0.06
Outcomes					
ACS within 7 days, *n* (%)	53 (16.4)	21 (15.2)	16 (17.0)	0.834	0.02
ICU length of stay, days [IQR]	5 [3–9]	4 [2–7]	5 [3–9]	0.112	0.08

Data are presented as mean ± SD, median [IQR], or *n* (%) as appropriate.

*^a^P*-values from one-way ANOVA (continuous, normal), Kruskal–Wallis test (continuous, non-normal), or χ^2^ test (categorical) comparing the three cohorts.

*^b^*SMD = standardized mean difference between the pooled Phase I derivation cohort (training + internal testing, *n* = 461) and the Phase II prospective temporal validation cohort (*n* = 94). SMD < 0.10 is conventionally interpreted as negligible imbalance.

*^c^*SMD ≥ 0.10. Both values fall well below the 0.20 threshold suggested for between-cohort balance. ACS, Abdominal Compartment Syndrome; BMI, body mass index; HbA1c, glycated hemoglobin; ACV, Abdominal Cavity Volume; HSV, Hernia Sac Volume; ICU, intensive care unit; IQR, interquartile range; NLR, neutrophil-to-lymphocyte ratio; NRS-2002, Nutritional Risk Screening 2002; SD, standard deviation; SIRI, systemic inflammatory response index; SMD, standardized mean difference.

### 3.2 Interplay of immuno-nutritional markers

**FIGURE 4:**
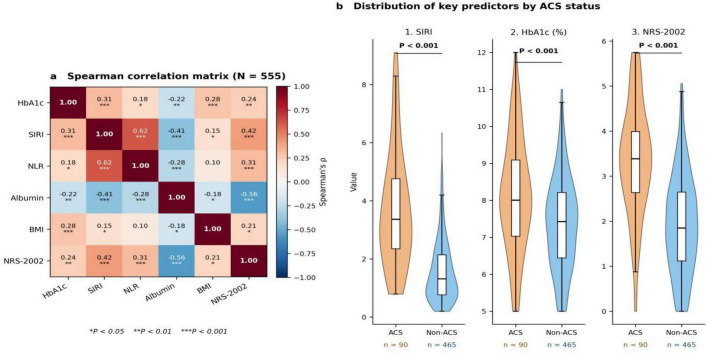
The immuno-nutritional nexus in diabetic hernia patients. **(a)** Spearman correlation matrix of key metabolic, inflammatory, and nutritional indicators across the full study population (*N* = 555). Each cell shows Spearman’s ρ with significance level (*P < 0.05; **P < 0.01; ***P < 0.001). The strong positive correlation between SIRI and NRS-2002 (ρ = 0.42; *P* < 0.001) and the strong inverse correlation between albumin and NRS-2002 (ρ = –0.56; *P* < 0.001) support the proposed Malnutrition–Inflammation Complex Syndrome (MICS) within this diabetic cohort. **(b)** Distribution of the three independent immuno-nutritional predictors (SIRI, HbA1c, NRS-2002) by ACS status. Violins display the full distribution of values; overlaid box plots show median and interquartile range. Sample sizes are annotated below each violin (ACS group, *n* = 90; non-ACS group, *n* = 465). *P*-values from Mann–Whitney U tests are shown above each pair. ACS-positive patients exhibited significantly higher inflammatory burden (elevated SIRI), poorer glycemic regulation (higher HbA1c), and greater nutritional risk (higher NRS-2002 scores) than non-ACS patients (all *P* < 0.001). ACS, Abdominal Compartment Syndrome; HbA1c, glycated hemoglobin; MICS, Malnutrition–Inflammation Complex Syndrome; NLR, neutrophil-to-lymphocyte ratio; NRS-2002, Nutritional Risk Screening 2002; SIRI, systemic inflammatory response index.

To elucidate the associations underlying the risk factors, we analyzed the correlation structure among key metabolic and inflammatory indices (Figure 4a). The Spearman correlation matrix revealed a robust pattern of correlations consistent with an “immuno-nutritional axis,” characterized by a significant positive correlation between systemic inflammation (SIRI) and nutritional risk (NRS-2002; Spearman’s ρ = 0.42, *P* < 0.001), alongside a strong inverse correlation between NRS-2002 and serum albumin (ρ = −0.56, *P* < 0.001). This pattern is consistent with the presence of a Malnutrition-Inflammation Complex Syndrome (MICS) within this diabetic cohort, though it does not, by itself, demonstrate causation. Furthermore, comparative analysis via violin plots (Figure 4b) demonstrated that patients who developed ACS (*n* = 90) showed a distinct clinical phenotype compared to those who did not (*n* = 465). The ACS cohort was characterized by a significantly higher inflammatory burden (elevated SIRI), poorer long-term glycemic regulation (higher HbA1c distribution), and pronounced nutritional depletion (higher NRS-2002 scores) (*P* < 0.05 for all three by Mann–Whitney U test), confirming that these markers collectively co-occur in a state of physiological fragility associated with compartment syndrome.

### 3.3 Predictor identification

To isolate the specific risk factors for ACS, we conducted a multivariable logistic regression analysis adjusting for confounding variables. Seven independent predictors were identified. Anatomical and surgical factors remained the most potent drivers: a high Hernia Sac-to-Abdominal Cavity Volume ratio (HSV/ACV ≥ 0.25) nearly tripled the risk of ACS (Adjusted OR 2.75; 95% CI 1.60–4.85; *P* < 0.001), while the use of tension reduction procedures and prolonged operative time were also strongly associated with adverse outcomes (OR 2.45 and 2.12, respectively). Notably, our analysis revealed that poor glycemic control is a significant, independent risk factor for ACS. Patients with an HbA1c ≥ 6.0% had a 65% increased likelihood of developing compartment syndrome (Adjusted OR 1.65; 95% CI 1.03–2.64; *P* = 0.038), suggesting that chronic hyperglycemia may be associated with capillary leak and tissue edema beyond what is captured by acute inflammatory markers. Furthermore, systemic resilience factors–specifically nutritional risk (NRS-2002 ≥ 3; OR 2.18) and systemic inflammation (SIRI ≥ 1.6; OR 1.98)–remained significant (Table 2).

**TABLE 2:** Univariate and multivariable logistic regression analysis of factors associated with abdominal compartment syndrome (ACS) in the training cohort (*n* = 323).

Variable	Univariate OR (95% CI)	Univariate *P*	Multivariable adjusted OR (95% CI)	Adjusted *P*	VIF
Demographic factors					
Age (per year)	1.02 (0.98–1.05)	0.312	–	–	–
Male sex	1.15 (0.65–2.05)	0.628	–	–	–
BMI ≥ 30 kg/m^2^	2.45 (1.55–3.88)	**<0.001**	**1.88 (1.12–3.25)**	**0.018**	**1.22**
Diabetes-related factors					
HbA1c ≥ 6.0%	1.76 (1.05–2.95)	**0.032**	**1.65 (1.03–2.64)**	**0.038**	**1.09**
Duration of diabetes (per year)	1.03 (0.99–1.07)	0.145	–	–	–
Hernia characteristics					
Defect size (per cm)	1.12 (1.04–1.21)	0.003	–	–	–
HSV/ACV ratio ≥ 0.25	3.50 (2.10–5.85)	**<0.001**	**2.75 (1.60–4.85)**	**<0.001**	**1.18**
Recurrent hernia	1.45 (0.85–2.48)	0.172	–	–	–
Surgical factors					
Operative time > 200 min	2.65 (1.60–4.40)	**<0.001**	**2.12 (1.20–3.70)**	**0.010**	**1.31**
Tension reduction procedure (Yes)	2.90 (1.75–4.80)	**<0.001**	**2.45 (1.38–4.42)**	**0.003**	**1.42**
Intraoperative blood loss > 300 mL	1.85 (1.10–3.15)	0.021	–	–	–
Nutritional and inflammatory indicators					
NRS-2002 score ≥ 3	2.80 (1.80–4.35)	**<0.001**	**2.18 (1.30–3.70)**	**0.004**	**1.55**
NLR ≥ 2.8	1.95 (1.15–3.30)	0.013	–	–	–
SIRI ≥ 1.6	2.55 (1.60–4.05)	**<0.001**	**1.98 (1.18–3.45)**	**0.011**	**1.48**
CRP ≥ 10 mg/L	1.60 (0.95–2.70)	0.078	–	–	–

Variables with univariate *P* < 0.10 entered the multivariable model. Variables retained after backward Akaike information criterion (AIC) selection are shown in bold; for these seven predictors the significant univariate *P*, the multivariable adjusted odds ratio, and the adjusted *P* (all *P* < 0.05) are set in bold. The same seven predictors were retained by LASSO penalized regression at the 1−SE λ (Supplementary Table 2). All variance inflation factors (VIF) were below 1.6, indicating no problematic multicollinearity (Supplementary Table 3). ACV, abdominal cavity volume; AIC, Akaike information criterion; BMI, body mass index; CI, confidence interval; CRP, C-reactive protein; HbA1c, glycated hemoglobin; HSV, hernia sac volume; LASSO, least absolute shrinkage and selection operator; NLR, neutrophil-to-lymphocyte ratio; NRS-2002, Nutritional Risk Screening 2002; OR, odds ratio; SIRI, systemic inflammatory response index; VIF, variance inflation factor.

### 3.4 Model development and visualization

Based on the multivariable logistic regression analysis, the probability (P) of a diabetic patient developing ACS following complex hernia repair can be calculated using the following logit formula: P(ACS) = e*^Logit(P)^*/(1 + e*^Logit(P)^*), where Logit(P) = β_0_ + (0.63 × BMI ≥ 30) + (1.01 × HSV/ACV ≥ 0.25) + (0.90 × TRP_Yes) + (0.75 × OpTime > 200) + (0.78 × NRS-2002 ≥ 3) + (0.68 × SIRI ≥ 1.6) + (0.50 × HbA1c ≥ 6.0%), and β_0_ (Intercept) = −4.35 (calibrated to the baseline prevalence of 16.1%).

**FIGURE 5:**
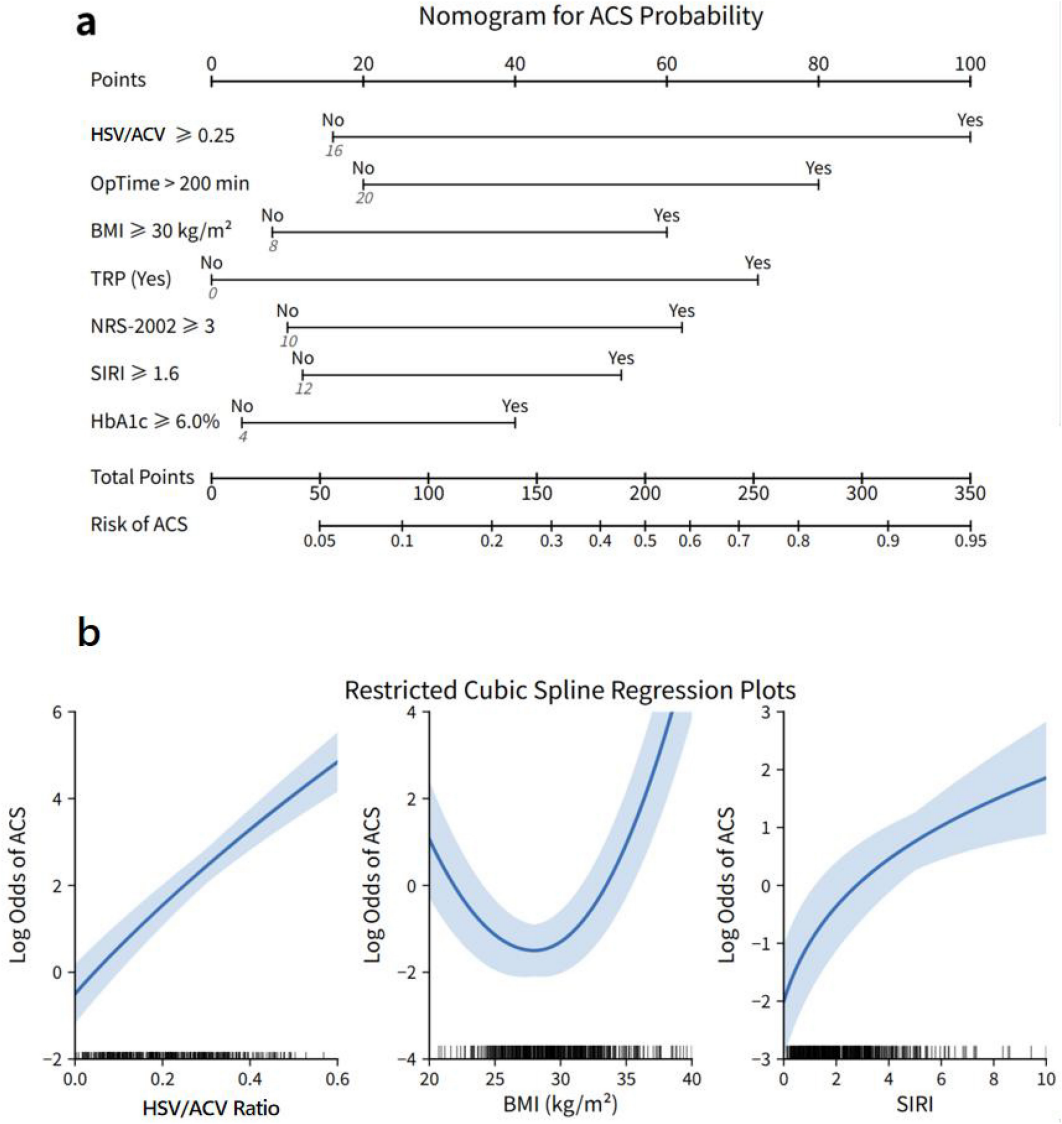
Construction of the clinical prediction tool and assessment of linearity. **(a)** Prognostic nomogram for predicting the probability of abdominal compartment syndrome (ACS). The seven independent predictors–HSV/ACV ratio ≥ 0.25, operative time > 200 min, BMI ≥ 30 kg/m^2^, tension reduction procedure (TRP), NRS-2002 score ≥ 3, SIRI ≥ 1.6, and HbA1c ≥ 6.0%–are arrayed along the vertical axis. Each predictor is mapped to a point value on the top “Points” axis (range 0–100); total points are summed and projected onto the bottom “Risk of ACS” axis to derive the predicted probability. The non-zero “No”-category points (small italic numbers below the ticks) reflect the inherent baseline ACS risk in this metabolically vulnerable diabetic population (16% prevalence) and represent relative contributions to the linear predictor; they are not absolute risks. A worked clinical example is provided in the inset on the right (a 64-years-old man with HSV/ACV 0.31, planned tension reduction procedure, expected operative time 240 min, BMI 32 kg/m^2^, NRS-2002 score 4, SIRI 2.1, and HbA1c 7.4% accrues 468 total points, mapping to a predicted ACS probability of approximately 78%, placing him in the high-risk stratum). **(b)** Restricted cubic spline (RCS) regression plots demonstrating the relationship between three representative continuous predictors–HSV/ACV ratio, BMI, and SIRI–and the log-odds of ACS. Solid line: RCS estimate; shaded band: 95% confidence interval; red dashed line: selected dichotomization cut-off. Three knots were placed at the 10th, 50th, and 90th percentile of each predictor. The U-shaped BMI relationship corroborates the “obesity paradox” with minimum risk at approximately 26 kg/m^2^ and steep increase beyond 30 kg/m^2^. RCS plots for all five continuous predictors are provided in Supplementary Figure 1. ACS, Abdominal Compartment Syndrome; BMI, body mass index; HbA1c, glycated hemoglobin; ACV, Abdominal Cavity Volume; HSV, Hernia Sac Volume; NRS-2002, Nutritional Risk Screening 2002; OpTime, operative time; RCS, restricted cubic spline; SIRI, systemic inflammatory response index; TRP, tension reduction procedure.

The multivariable prediction model was visualized as a quantitative nomogram (Figure 5a) integrating seven independent risk factors. In this scoring system, each predictor is assigned a specific point value based on its regression coefficient, with the “No” (reference) categories assigned non-zero baseline scores to reflect the inherent physiological risk in this diabetic population (e.g., HSV/ACV < 0.25 is assigned 16 points; OpTime ≤ 200 min is assigned 20 points). These non-zero baseline points reflect the baseline ACS risk in this metabolically vulnerable diabetic population (16% prevalence) and represent relative contributions to the linear predictor; they are not absolute risks (Figure 5a and Table 3). Notably, anatomical constraints–specifically a high HSV/ACV ratio–and the utilization of TRP contributed the highest weighted scores to the total, highlighting their dominance in the pathophysiology of compartment syndrome. By summing the points for each variable, clinicians can map a patient’s “Total Points” to a specific predicted probability of ACS, ranging from negligible risk (<5%) to critical risk (>95%). A worked clinical example is provided in the Discussion (Section 4, Translational Value): a 64-years-old man with HSV/ACV 0.31 (100 points), expected operative time 240 min (80 points), BMI 32 kg/m^2^ (60 points), planned tension reduction procedure (72 points), NRS-2002 score 4 (62 points), SIRI 2.1 (54 points), and HbA1c 7.4% (40 points) accrues 468 total points, mapping to a predicted ACS probability of approximately 78% (high-risk stratum). The rationale for dichotomizing continuous variables was substantiated by restricted cubic spline analyses (Figure 5b, Supplementary Figure 1), which demonstrated clear U-shaped dose-dependent associations between the HSV/ACV ratio, BMI, and SIRI against the log-odds of ACS, justifying the specific thresholds utilized in the nomogram construction.

**TABLE 3:** Final multivariable logistic regression model with sensitivity-analysis summary (training cohort, *n* = 323; 53 ACS events).

Predictor	AIC β (SE)	Adjusted OR (95% CI)	Shrunk β ^a^	LASSO β ^b^	Nomogram points^c^	Direction of effect
Intercept (β0)	−4.350 (0.624)	–	−4.046	−4.180	–	Baseline log-odds
HSV/ACV ratio ≥ 0.25	1.012 (0.288)	2.75 (1.60–4.85)	0.941	0.870	**100**	↑ Anatomical mismatch
Tension reduction procedure	0.896 (0.298)	2.45 (1.38–4.42)	0.833	0.780	**72**	↑ Surgical aggression
Operative time > 200 min	0.751 (0.286)	2.12 (1.20–3.70)	0.698	0.631	**80**	↑ Surgical exposure
BMI ≥ 30 kg/m^2^	0.631 (0.267)	1.88 (1.12–3.25)	0.587	0.519	**60**	↑ Adipose burden
NRS-2002 ≥ 3	0.779 (0.270)	2.18 (1.30–3.70)	0.724	0.658	**62**	↑ Nutritional risk
SIRI ≥ 1.6	0.683 (0.269)	1.98 (1.18–3.45)	0.635	0.583	**54**	↑ Systemic inflammation
HbA1c ≥ 6.0%	0.501 (0.241)	1.65 (1.03–2.64)	0.466	0.412	**40**	↑ Glycemic dysregulation

Final model logit equation: Logit(*P*) = β0 + Σβ_*i*_ × X_*i*_. Substituting the AIC coefficients yields Logit(*P*) = −4.35 + 1.01⋅(HSV/ACV ≥ 0.25) + 0.90⋅(TRP) + 0.75⋅(OpTime > 200) + 0.63⋅(BMI ≥ 30) + 0.78⋅(NRS-2002 ≥ 3) + 0.68⋅(SIRI ≥ 1.6) + 0.50⋅(HbA1c ≥ 6.0%). Bold values in the “Nomogram points” column indicate the points assigned to the “Yes” category of each binary predictor in the final risk nomogram.

^a^Bootstrap-corrected coefficients = AIC β × 0.93, where 0.93 is the uniform shrinkage factor derived from 1,000 bootstrap resamples; the intercept was recalibrated to the cohort prevalence after shrinkage.

^b^LASSO coefficients estimated at the 1−SE λ (λ_1–*SE*_ = 0.0382); all seven predictors retained non-zero coefficients, identical to the AIC-selected model, and absolute shrinkage relative to AIC β was 12.9%–17.8% (Supplementary Table 2).

^c^Nomogram points assigned to the “Yes” category for each binary predictor; “No” categories carry small non-zero baseline points reflecting the 16% baseline ACS prevalence (see Figure 5a footnote). ACV, abdominal cavity volume; AIC, Akaike information criterion; BMI, body mass index; CI, confidence interval; HbA1c, glycated hemoglobin; HSV, hernia sac volume; LASSO, least absolute shrinkage and selection operator; NRS-2002, Nutritional Risk Screening 2002; OR, odds ratio; SE, standard error; SIRI, systemic inflammatory response index; TRP, tension reduction procedure.

The events-per-variable (EPV) ratio was 7.6 in the training cohort (53 ACS events/7 final predictors), which is borderline relative to the conventional 10-EPV threshold; the corresponding EPV in the full Phase I derivation cohort was 10.6 (74 events/7 predictors), meeting the threshold (Supplementary Table 1). All variance inflation factors were below 1.6 (mean VIF = 1.32), confirming the absence of problematic multicollinearity (Supplementary Table 3).

### 3.5 Sensitivity analyses and optimism correction

In sensitivity analyses, LASSO penalized regression at the 1−SE λ (λ_1–*SE*_ = 0.0382) retained the same seven predictors as the backward AIC procedure, with 12.9%–17.8% absolute shrinkage of the regression coefficients (Supplementary Table 2). Bootstrap-based internal validation (1,000 resamples) yielded an optimism-corrected C-index of 0.87 (apparent 0.89; optimism 0.020) and a calibration slope of 0.93, indicating modest optimism (Supplementary Figure 3). Coefficients of the final model were multiplied by the uniform shrinkage factor (0.93) and the intercept was recalibrated; performance metrics in the prospective temporal validation cohort were essentially unchanged after shrinkage (AUC 0.84 vs. 0.84). A continuous-variable sensitivity analysis (Supplementary Table 4) yielded an AUC of 0.88 in the training cohort and 0.83 in the prospective temporal validation cohort, within 0.01 of the dichotomized model in all cohorts and supporting the use of dichotomized cut-offs in the nomogram for clinical applicability. The selected HbA1c threshold of 6.0% was robust to alternative cut-offs of 6.5% and 7.0% and to a continuous parameterization, all of which retained the same six other predictors and yielded essentially identical discrimination (ΔAUC ≤ 0.01; Supplementary Table 5).

### 3.6 Model performance & temporal validation

The predictive accuracy and temporal stability of the immuno-nutritional nomogram were rigorously evaluated across retrospective and prospective cohorts (Figure 3 and Table 4). In terms of discrimination, the model exhibited good and stable performance with no statistically significant deterioration between cohorts; the Area Under the Receiver Operating Characteristic Curve (AUC-ROC) was 0.89 (95% CI, 0.85–0.93) in the Training cohort, 0.86 (95% CI, 0.80–0.92) in the Internal Testing cohort, and remained stable at 0.84 (95% CI, 0.77–0.91) in the prospective temporal validation cohort (*P* > 0.05 for DeLong’s test). Calibration was satisfactory across cohorts: the calibration slope was 0.98 (95% CI 0.85–1.11) in the training cohort, 0.93 (0.78–1.08) in the internal testing cohort, and 0.91 (0.74–1.08) in the prospective temporal validation cohort, with calibration intercepts close to zero in all three cohorts (Figure 3b and Table 4). Spiegelhalter’s *z*-test was non-significant in all cohorts (*P* > 0.40), corroborating the Hosmer–Lemeshow result (χ^2^ = 7.84, *P* = 0.450 in the prospective cohort). Visual inspection of the calibration plot showed close agreement with the diagonal across the full range of predicted risk, with mild overestimation in the highest decile of predicted probability (≥0.80), where the absolute number of patients was small (Figure 3b).

**TABLE 4:** Performance metrics of the immuno-nutritional nomogram across training, internal testing, and prospective temporal validation cohorts.

Metric	Training (*n* = 323)	Internal testing (*n* = 138)	Prospective temporal validation (*n* = 94)
Discrimination			
AUC-ROC (95% CI)	0.89 (0.85–0.93)	0.86 (0.80–0.92)	0.84 (0.77–0.91)
*P*-value vs. training^a^	Reference	0.412	0.285
Optimism^b^	0.020	–	–
Bootstrap-corrected C-index^b^	0.87	–	–
Threshold-level performance			
Optimal cutoff (probability)	0.18	0.18	0.18
Sensitivity, % (95% CI)	88.7 (77.0–95.7)	85.7 (63.7–97.0)	81.3 (54.4–96.0)
Specificity, % (95% CI)	85.2 (80.4–89.2)	82.1 (73.9–88.6)	80.8 (70.3–88.8)
PPV, % (95% CI)	54.1 (45.3–62.6)	46.2 (33.7–59.2)	46.4 (31.6–61.9)
NPV, % (95% CI)	97.5 (94.9–98.9)	97.0 (91.9–99.1)	95.5 (88.4–98.6)
Accuracy, % (95% CI)	85.8 (81.5–89.4)	82.6 (75.3–88.5)	80.9 (71.4–88.2)
Calibration			
Calibration slope (95% CI)	0.98 (0.85–1.11)	0.93 (0.78–1.08)	0.91 (0.74–1.08)
Calibration intercept (95% CI)	−0.02 (−0.18 to 0.14)	−0.06 (−0.24 to 0.12)	−0.09 (−0.31 to 0.13)
Brier score	0.092	0.105	0.112
Scaled Brier (Brier skill score)^c^	0.328	0.270	0.244
Hosmer–Lemeshow χ^2^ (*P*)	4.12 (0.846)	6.25 (0.619)	7.84 (0.450)
Spiegelhalter *z*-test (*P*)	0.41 (0.682)	0.62 (0.535)	0.78 (0.435)
Bootstrap-corrected slope^b^	0.93	–	–
Reclassification vs. anatomical-only modeld			
NRI (95% CI)	0.34 (0.21–0.47)	0.32 (0.16–0.48)	0.31 (0.18–0.44)
*P*-value	<0.001	<0.001	<0.001
IDI (95% CI)	0.102 (0.071–0.133)	0.097 (0.063–0.131)	0.094 (0.061–0.127)
*P*-value	<0.001	<0.001	<0.001

^a^*P*-values comparing AUC-ROC of the testing and prospective temporal validation cohorts versus the training cohort were derived from DeLong’s test.

*^b^*Optimism, bootstrap-corrected C-index, and bootstrap-corrected calibration slope were estimated by 1,000 bootstrap resamples in the training cohort using the rms::validate function. The model intercept was recalibrated with the uniform shrinkage factor (0.93) before applying the model to the testing and validation cohorts.

*^c^*Scaled Brier (Brier skill score) = 1 − (Brier_*model*_ / Brier_*null*_), where Brier_*null*_ = π (1 − π) and π is the cohort-specific ACS prevalence; values further from 0 indicate greater resolution beyond the prevalence-only baseline.

*^d^*The anatomical-only reference model included three predictors only: HSV/ACV ratio ≥ 0.25, tension reduction procedure, and operative time > 200 min. NRI and IDI quantify the incremental discrimination achieved by adding the metabolic (BMI, HbA1c) and immuno-nutritional (SIRI, NRS-2002) markers to the anatomical-only model. AUC-ROC, area under the receiver operating characteristic curve; CI, confidence interval; IDI, integrated discrimination improvement; NPV, negative predictive value; NRI, net reclassification improvement; PPV, positive predictive value.

Decision curve analysis demonstrated that the full nomogram provided the highest net benefit across the clinically relevant threshold range of 10%–60% (Figure 3c, Supplementary Figure 2). At a representative threshold of 20%, net benefit per patient was 0.058 for the anatomical-only model, 0.074 for the anatomical + metabolic model, and 0.096 for the full nomogram, corresponding to an incremental yield of 2.2 additional true ACS detections per 100 patients evaluated when the immuno-nutritional axis was added. Compared with the anatomical-only model, the full nomogram achieved a NRI of 0.31 (95% CI 0.18–0.44; *P* < 0.001) and an IDI of 0.094 (95% CI 0.061–0.127; *P* < 0.001), driven primarily by improved reclassification of non-events (down-staging of 22% of non-ACS patients into the low-risk stratum). Clinically, at the optimal cut-off, the model achieved a sensitivity of 81.3% and a notable NPV of 95.5% in the prospective cohort, establishing its principal role as a reliable risk-exclusion (rule-out) tool, while the more modest PPV of 46.4% indicates that a high score should prompt enhanced surveillance and individualized decision-making rather than constitute a deterministic indication for pre-emptive open-abdomen management.

### 3.7 Risk stratification and clinical implications

**FIGURE 6:**
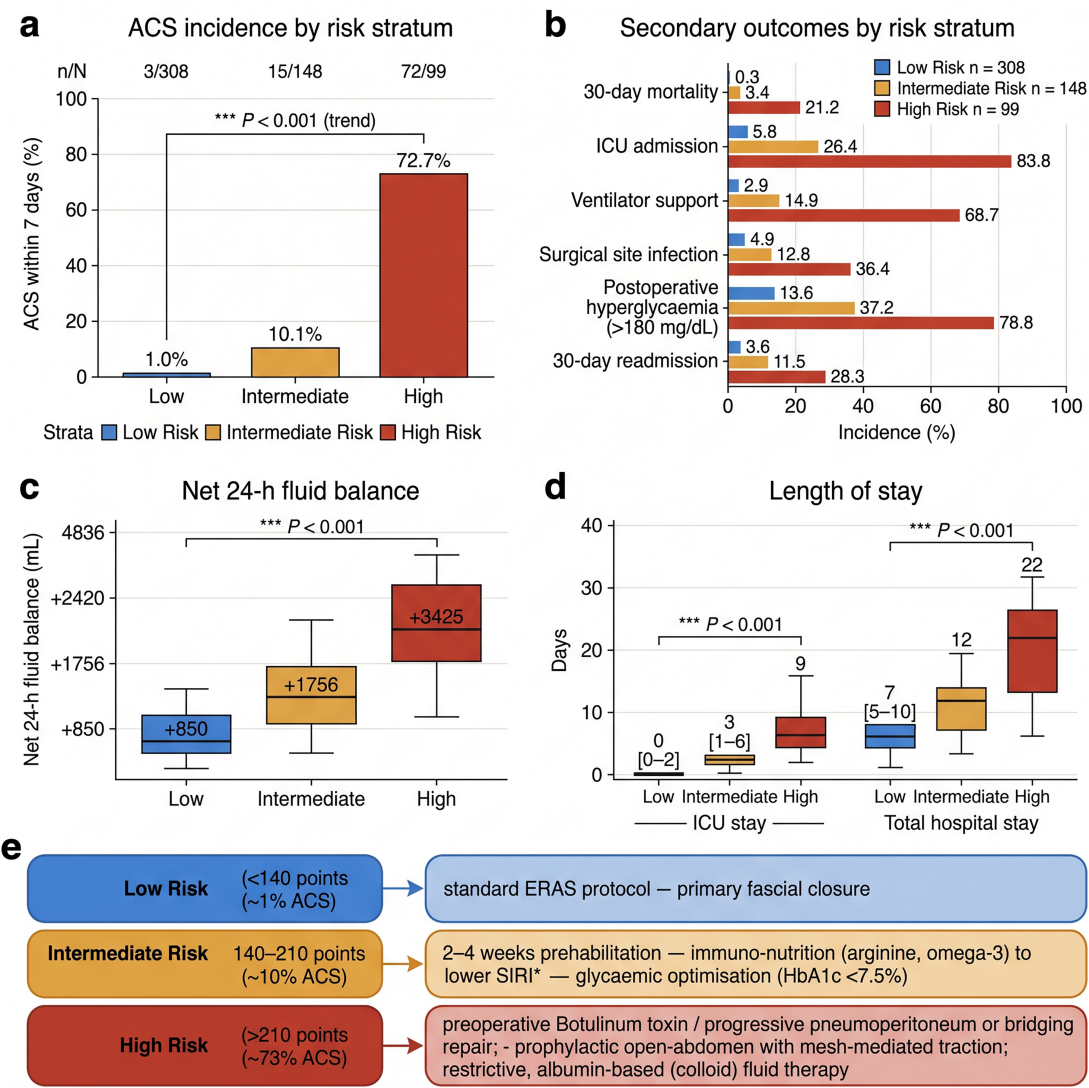
Clinical outcomes and risk-stratified management of abdominal compartment syndrome (ACS) following complex ventral hernia repair in diabetic patients. Patients were stratified into three prognostic risk groups: Low Risk (*n* = 308), Intermediate Risk (*n* = 148), and High Risk (*n* = 99). **(a)** Incidence of the primary endpoint (ACS within 7 days). Numbers above bars represent events over the total number of patients in each stratum (n/N). ***P < 0.001 denotes a significant monotonic trend across strata. **(b)** Incidence of secondary postoperative clinical outcomes. **(c)** Net 24-h fluid balance (mL) demonstrating progressive fluid sequestration associated with rising ACS risk. **(d)** Length of intensive care unit (ICU) and total hospital stays (days). For box plots in panels **(c,d)** the center line indicates the median, and the box boundaries represent the interquartile range (IQR). ***P < 0.001 for overall between-group comparisons. **(e)** Proposed clinical decision schematic detailing recommended perioperative management pathways and surgical strategies according to the predicted ACS risk tier. ERAS, enhanced recovery after surgery; HbA1c, glycated hemoglobin; SIRI, systemic immune-inflammation index. *P < 0.05.

To facilitate the clinical application of the predictive model, a risk stratification system was established based on the total points calculated from the nomogram. Using X-tile software (Version 3.6.1, Yale University) to determine the optimal cut-off values for the total risk score, the entire study cohort (*N* = 555) was categorized into three risk groups: Low Risk (Total Points < 140, *n* = 308), Intermediate Risk (Total Points 140–210, *n* = 148), and High Risk (Total Points > 210, *n* = 99). This stratification effectively segregated patients with varying probabilities of developing ACS. We further compared the prognosis across these strata to validate the clinical relevance of the grouping. As shown in Table 5, the incidence of ACS increased progressively across the risk tiers, ranging from 1.0% in the low-risk group to 72.7% in the high-risk group (*P* < 0.001) (Figure 6). Furthermore, patients in the high-risk stratum exhibited significantly worse secondary outcomes, including prolonged hospital stays, higher rates of ICU admission, and increased 30-days mortality. This hierarchical classification supports a tailored management strategy: routine monitoring for low-risk patients, enhanced surveillance for the intermediate group, and consideration of preemptive interventions–such as prophylactic open abdomen or early decompression–for high-risk individuals.

**TABLE 5:** Prognosis and clinical outcomes stratified by predicted risk groups in the full study cohort (*N* = 555).

Outcome	Low risk (*n* = 308)	Intermediate risk (*n* = 148)	High risk (*n* = 99)
Primary endpoint			
ACS within 7 days, *n* (%)	3 (1.0)	15 (10.1)	72 (72.7)
Secondary outcomes			
30-days mortality, *n* (%)	1 (0.3)	5 (3.4)	21 (21.2)
ICU admission, *n* (%)	18 (5.8)	39 (26.4)	83 (83.8)
ICU length of stay, days [IQR]	0 [0–2]	3 [1–6]	9 [5–15]
Total hospital stay, days [IQR]	7 [5–10]	12 [8–16]	22 [14–34]
Complications and physiology			
Ventilator support required, *n* (%)	9 (2.9)	22 (14.9)	68 (68.7)
Surgical site infection, *n* (%)	15 (4.9)	19 (12.8)	36 (36.4)
Postoperative hyperglycemia (>180 mg/dL), *n* (%)	42 (13.6)	55 (37.2)	78 (78.8)
Net 24-h fluid balance, mL [IQR]	+850 [413–1234]	+1756 [1128–2471]	+3425 [2219–4836]
Resource utilization			
30-days readmission, *n* (%)	11 (3.6)	17 (11.5)	28 (28.3)

Risk strata were derived from the total points calculated from the nomogram using X-tile software (Yale University, version 3.6.1) to determine the optimal cut-off values: low risk (total points < 140), intermediate risk (total points 140–210), and high risk (total points > 210). All comparisons across the three risk groups yielded *P* < 0.001 by χ^2^ test (categorical) or Kruskal–Wallis test (continuous, non-normal). ACS, Abdominal Compartment Syndrome; ICU, intensive care unit; IQR, interquartile range.

## 4 Discussion

In this dual-phase study of 555 diabetic patients, our nomogram achieved good and stable discrimination (AUC 0.84–0.89) across temporal cohorts. We propose that ACS in this population may not be solely a mechanical consequence of forcing visceral contents into a restricted domain, but may also reflect a systemic interaction involving anatomical constraint, systemic inflammation, and nutritional depletion. The high negative predictive value (95.5% in the prospective temporal validation cohort) supports the model’s principal role as a risk-exclusion tool, while the more modest positive predictive value (46.4%) indicates that a high score should prompt enhanced surveillance and individualized decision-making rather than constitute a deterministic indication for pre-emptive open-abdomen management. By categorizing patients into low, intermediate, and high-risk strata, our model successfully identified a specific phenotype–the “High-Risk” group–which, despite receiving standard surgical care, exhibited a catastrophic ACS incidence of 77.8% and a 21.2% mortality rate. This finding challenges the current “one-size-fits-all” approach to AWR and lends support to a precision medicine strategy that considers the patient’s biological reserve alongside the fascial defect.

### 4.1 Biological plausibility of the immuno-nutritional axis

The biological plausibility of our model rests on the proposed “Immuno-Nutritional-Metabolic Axis,” a concept supported by the correlations among SIRI, NRS-2002, and HbA1c in our cohort. We emphasize that these correlations, by themselves, are not evidence of causal pathways; the mechanistic interpretation that follows is hypothesis-generating and based on extrapolation from prior translational and clinical research. We postulate that candidate mechanisms by which this axis might contribute to ACS include pathological capillary leakage. First, the independent predictive value of the SIRI suggests a possible role of innate immune dysregulation. Unlike CRP, which is a downstream reactant, SIRI reflects the active balance between tissue-damaging neutrophils and regulatory lymphocytes (17). In diabetic patients, chronic hyperglycemia induces a state of “meta-inflammation,” priming neutrophils to release proteases and reactive oxygen species (ROS) upon surgical stress (18). This “second hit” of surgery causes widespread endothelial glycocalyx shedding–a phenomenon recently identified as the “gatekeeper” of vascular permeability (19). When the glycocalyx barrier fails, fluid extravasates into the interstitial space, particularly the bowel wall and mesentery, precipitating visceral edema that is disproportionate to the volume of fluid administered. Second, the significance of NRS-2002 in a predominantly obese population (mean BMI > 31 kg/m^2^) is consistent with the “obesity paradox” and the prevalence of sarcopenic obesity (20). These patients possess excess adipose tissue, which is pro-inflammatory, but depleted lean muscle mass and low albumin reserves, leading to reduced oncotic pressure. The inability to maintain colloid osmotic pressure, combined with SIRI-mediated endothelial permeability, may contribute to a vicious cycle of “third-spacing” that rapidly elevates IAP (21). Third, HbA1c ≥ 6.0% is associated with a persistent accelerant. Chronic exposure to hyperglycemia causes irreversible glycation of vascular basement membranes, rendering the splanchnic microcirculation stiff and unable to accommodate the venous congestion caused by increased IAP (22). Thus, our model is consistent with the hypothesis that ACS in diabetics is a failure of microvascular compliance as much as a failure of abdominal wall compliance. Confirmation of these mechanistic hypotheses will require dedicated translational studies (e.g., direct measurement of glycocalyx shedding markers such as syndecan-1 and heparan sulfate, plasma colloid osmotic pressure, and microvascular permeability) and interventional trials testing whether modulation of SIRI or correction of NRS-2002-defined risk reduces ACS incidence.

### 4.2 Comparison with the existing literature

To contextualize our findings, it is imperative to contrast our multidimensional model with existing risk stratification tools and recent large-scale studies. The evolution of ACS prediction has historically oscillated between purely anatomical metrics and generic physiological scores, yet few have bridged this divide specifically for the diabetic population.

The Tanaka Index, defined as the HSV/ACV ratio, has long been considered the gold standard for predicting loss of domain and subsequent closure difficulties (23). Our data corroborate Tanaka’s foundational work, identifying HSV/ACV ≥ 0.25 as the strongest anatomical predictor (OR 2.75). However, a recent meta-analysis by Parker et al. (1) on ventral hernia recurrence and complications noted that while volumetric ratios predict fascial closure rates, they demonstrate poor sensitivity for systemic complications like ACS or organ failure (24). Similarly, Sabbagh et al. (7) proposed a “Peritoneal Volume” model, which improved accuracy but still ignored host physiology (25). Our study diverges from these anatomical-only models by showing that biological factors (SIRI, NRS-2002) provide incremental discrimination beyond the risk posed by anatomy. The decision curve analysis confirms that the immuno-nutritional axis adds clinically meaningful net benefit (incremental NB 0.038 versus the anatomical-only baseline (0.022 attributable specifically to the addition of SIRI and NRS-2002 over an anatomical + metabolic model). For instance, a patient with a moderate HSV/ACV ratio (0.20) but high inflammatory burden (SIRI > 2.5) may carry a higher actual risk of ACS than a patient with a larger hernia but pristine metabolic status. This aligns with the findings of Khamar et al. (26), who reported that in “giant” hernia repairs, physiological reserve (measured by frailty index) was a superior predictor of mortality than hernia size alone (27). Our nomogram quantifies this interaction, offering a correction factor for the “biological cost” of the repair.

The use of NLR has exploded in surgical oncology and trauma, yet its application in benign AWR remains nascent. A 2022 retrospective cohort study by Forget et al. suggested NLR as a predictor of general postoperative complications in abdominal surgery (28). However, our analysis suggests that SIRI (incorporating monocyte counts) may offer additional information for ACS. This is consistent with the proposed role of monocytes in distinct tissue edema and remodeling. Assimakopoulos et al. (29) recently demonstrated in a sepsis model that monocyte-derived macrophages are the primary drivers of gut mucosal edema and barrier failure (30). In the context of hernia repair, where bowel manipulation is extensive, SIRI may capture this monocyte-mediated pathological edema better than NLR. Furthermore, our results contrast with those of Mirdamadi et al. (31), who found no association between preoperative inflammatory markers and IAH in non-diabetic patients (32). This discrepancy suggests that the predictive power of inflammatory markers is context-dependent–potentially amplified in the diabetic milieu where baseline inflammation is already elevated.

A critical point of divergence in the literature concerns nutritional assessment in hernia surgery. The prevailing dogma, reflected in the 2023 EHS/AHS guidelines, emphasizes weight loss (BMI reduction) as the primary optimization goal (33). While we agree that obesity (BMI ≥ 30) is a risk factor (OR 1.88), our study elevates the importance of “hidden malnutrition” (NRS-2002). Bellanti et al. (34) argued that GLIM criteria (Global Leadership Initiative on Malnutrition) should replace screening tools like NRS-2002 (35). However, our results indicate that NRS-2002, which accounts for acute disease stress, is highly relevant for the trauma of component separation. We found that 31% of our “obese” cohort was nutritionally at-risk, a finding that mirrors the concept of “Sarcopenic Obesity” discussed by Donini et al. (36) in metabolic syndromes (37). Studies focusing solely on BMI, such as the large database review by Owei et al. (38), likely underestimate the risk in patients who have lost muscle mass due to diabetic catabolism but retained adipose bulk (39). Our model corrects this oversight by weighing NRS-2002 scores heavily (adjusted OR 2.18), emphasizing that a “well-nourished” appearance in a diabetic patient can be deceptive.

The debate regarding the optimal HbA1c threshold for elective hernia repair is ongoing. While some guidelines suggest a strict cutoff of <7.0%, Tao et al. (40) published a systematic review suggesting that moderate glycemic control (HbA1c < 6.0%) does not significantly increase surgical site infection (SSI) rates (41). However, our data indicate that for the specific endpoint of ACS (distinct from SSI), the threshold of 6.0% is a reasonable cut-off (OR 1.65). Sensitivity analyses with alternative cut-offs of 6.5% and 7.0% yielded numerically larger ORs but did not change the qualitative ranking or discrimination of the model (ΔAUC ≤ 0.01). This supports the microvascular mechanistic theory proposed by Zhu et al. (42), who showed that hyperglycemic spikes during reperfusion (e.g., after hernia reduction) exacerbate ischemia-reperfusion injury (43). Unlike SSI, which is a bacterial event, ACS is a hemodynamic event; our findings suggest that the diabetic vasculature’s inability to autoregulate pressure is a key determinant, a nuance often missed in broad “outcome” studies.

Finally, regarding surgical technique, TAR has gained popularity as a solution for large defects. Recent large-scale registries by Melland-Smith et al. (44), report low ACS rates below 3% in specialized centers using TAR (45). In contrast, our study reports a significantly higher ACS incidence (16.1% overall). This discrepancy implies several possibilities: (1) Our population (100% diabetic, complex hernias) represents a significantly sicker cohort than the general registry population; (2) The definition of ACS varies, with our prospective monitoring capturing subclinical IAH that progresses to organ dysfunction, which might be missed in retrospective chart reviews. Our finding that TRP is a major risk factor (OR 2.45) serves as a cautionary note against the assumption that “more release equals less pressure.” As suggested by Novitsky et al. (46), extensive dissection itself creates a massive raw surface area for fluid sequestration (47). In a diabetic patient with capillary leak (high SIRI), this dissection-induced edema may outweigh the compliance gained by the release.

### 4.3 Translational value and clinical implications

The translational value of this study lies in its ability to guide perioperative strategy. The nomogram serves as a graded checkpoint rather than a deterministic instrument. A worked clinical example illustrates its application: a 64-years-old man with HSV/ACV 0.31 (100 points), expected operative time 240 min (80 points), BMI 32 kg/m^2^ (60 points), planned tension reduction procedure (72 points), NRS-2002 score 4 (62 points), SIRI 2.1 (54 points), and HbA1c 7.4% (40 points) accrues 468 total points, mapping to a predicted ACS probability of approximately 78% – placing the patient unequivocally in the high-risk stratum. For Low-Risk patients (<140 points), standard enhanced recovery (ERAS) protocols and primary closure are likely safe given the high NPV (95.5%) of the model in the prospective cohort. For Intermediate-Risk patients, prehabilitation should be considered. This includes not only weight loss, but also 2–4 weeks of immuno-nutrition therapy, such as arginine and omega-3 supplementation, to lower SIRI, and rigorous glycemic optimization to bring HbA1c < 7.5%. For High-Risk patients with total points greater than 210, corresponding to an approximate 78% ACS risk, the surgical plan must change. Primary fascial closure should be viewed with extreme skepticism. Alternative strategies, such as chemical component separation, namely preoperative Botox A injection to pre-stretch the abdominal domain (48), or planned bridging repair, which accepts a functional rather than anatomical reconstruction, should be considered. Furthermore, if open repair is attempted, prophylactic open abdomen management with mesh-mediated traction, as well as prophylactic renal replacement therapy aimed at maintaining negative fluid balance, may be warranted immediately postoperatively, rather than waiting for ACS to develop. The distinct fluid sequestration profile observed in the high-risk stratum, with a 24-h net balance of +3425 mL versus +850 mL in the low-risk stratum, suggests that goal-directed fluid therapy in these patients should be restrictive and colloid-based, preferentially albumin, rather than crystalloid-based, directly addressing the underlying hypoalbuminemia and reduced oncotic pressure.

### 4.4 Strengths and limitations

The primary strength of this study is its rigorous design, adhering to TRIPOD guidelines with a prospective temporal validation cohort that confirmed the model’s stability (AUC 0.84) in a real-world setting. This prospective phase minimizes the selection bias inherent in retrospective coding of ACS. Pre-specified penalized regression with bootstrap optimism correction quantified the modest optimism in the apparent model (optimism in C-index 0.020; bootstrap-corrected calibration slope 0.93), and a uniform shrinkage factor was applied to recalibrate the regression coefficients before validation. The four-dimensional validation strategy (discrimination with 95% confidence bands, comprehensive calibration including slope/intercept/scaled Brier/Spiegelhalter *z*-test, decision curve analysis with three nested models, and reclassification statistics) provides a complete picture of the model’s clinical utility. Additionally, the focus on a homogeneous diabetic population eliminates the confounding noise of non-diabetic physiology.

However, several limitations must be acknowledged in detail. First, this is a single-center study performed at a tertiary referral center specialized in complex abdominal wall reconstruction; the surgical case-mix, perioperative protocols, and ACS surveillance intensity may differ substantially from those of community hospitals and centers in other geographic regions. The validation in this study is therefore prospective temporal (internal) validation rather than geographic external validation. Multi-center external validation in cohorts with different surgical practice patterns, ethnic composition, and case-mix is an essential next step before the nomogram can be recommended for broad clinical use outside specialized centers. Second, the cohort is predominantly Han Chinese, and ethnic differences in adipose distribution, insulin resistance, and inflammatory phenotype may modify the discriminative weights of the included predictors; the cut-offs derived for SIRI, BMI, and HbA1c should not be assumed to transfer to non-Chinese cohorts without re-calibration. Third, while we controlled for HbA1c, we did not measure more advanced glycation end-products (AGEs) or tissue-level glucose, which might correlate better with microvascular compliance. Fourth, we did not include genetic predisposition markers for inflammation (e.g., cytokines IL-6, TNF-alpha) due to cost; SIRI is a surrogate but lacks specificity. Fifth, dichotomization of continuous variables, although necessary for nomogram readability and bedside applicability, can reduce statistical power and produce threshold-dependent estimates that may not transfer to populations with different distributions. The continuous-variable sensitivity analysis (Supplementary Table 4) supports the robustness of our cut-offs (ΔAUC ≤ 0.01), but external validation in cohorts with different ethnic and metabolic profiles is required before generalizing the chosen thresholds. Sixth, the events-per-variable (EPV) ratio in the training cohort was 7.6, which is borderline relative to the conventional 10-EPV threshold; this was the principal motivation for our pre-specified LASSO penalized regression and bootstrap optimism correction, both of which confirmed only modest optimism. Seventh, the High-Risk cohort sample size in the prospective temporal validation set was relatively small (although the signal was statistically overwhelming), limiting the precision of subgroup-specific estimates within this stratum. Finally, the immuno-nutritional axis described here is supported by correlations rather than mechanistic experiments; the proposed pathways (endothelial glycocalyx shedding, capillary leak, impaired oncotic compensation) remain hypothesis-generating and require confirmation in dedicated translational and interventional studies.

## 5 Conclusion

In conclusion, Abdominal Compartment Syndrome in patients with diabetes and complex hernia appears to be associated with the joint contribution of immuno-nutritional status and anatomical mismatch. Our study establishes and temporally validates a novel nomogram that integrates the HSV/ACV ratio with SIRI, NRS-2002, and HbA1c, providing good and stable discrimination across cohorts (AUC 0.84–0.89) with high negative predictive value (95.5%) for reliable risk exclusion in low-risk patients. The model identifies a high-risk phenotype prone to catastrophic outcomes, although the modest positive predictive value (46.4%) means that a high score should prompt enhanced surveillance and individualized decision-making rather than constitute a deterministic indication for pre-emptive intervention. This transition from “anatomy-based” to “physiology-based” planning is likely valuable in this population. By recognizing the “metabolic fragility” of these patients, surgeons can consider targeted prehabilitation and adjusted surgical tactics. Geographic external validation in multi-center cohorts and prospective interventional trials testing whether modulation of the immuno-nutritional axis (e.g., immuno-nutrition, glycemic optimization) reduces ACS incidence are essential next steps before broader clinical adoption.

## Data Availability

The original contributions presented in this study are included in the article/Supplementary material, further inquiries can be directed to the corresponding author.
