# Supplementary material for: Synergistic impact of nutritional risk, glycemic control, and systemic inflammation on Abdominal Compartment Syndrome in diabetic patients following complex ventral hernia repair: a development and validation study
Source: Front Nutr. 2026 Jun 19;13:1786526. doi: 10.3389/fnut.2026.1786526 (PMC13328177; doi:10.3389/fnut.2026.1786526)
Supplement: Supplementary file 1 [file Data_Sheet_1.pdf]

Supplementary Figure Titles and Legends

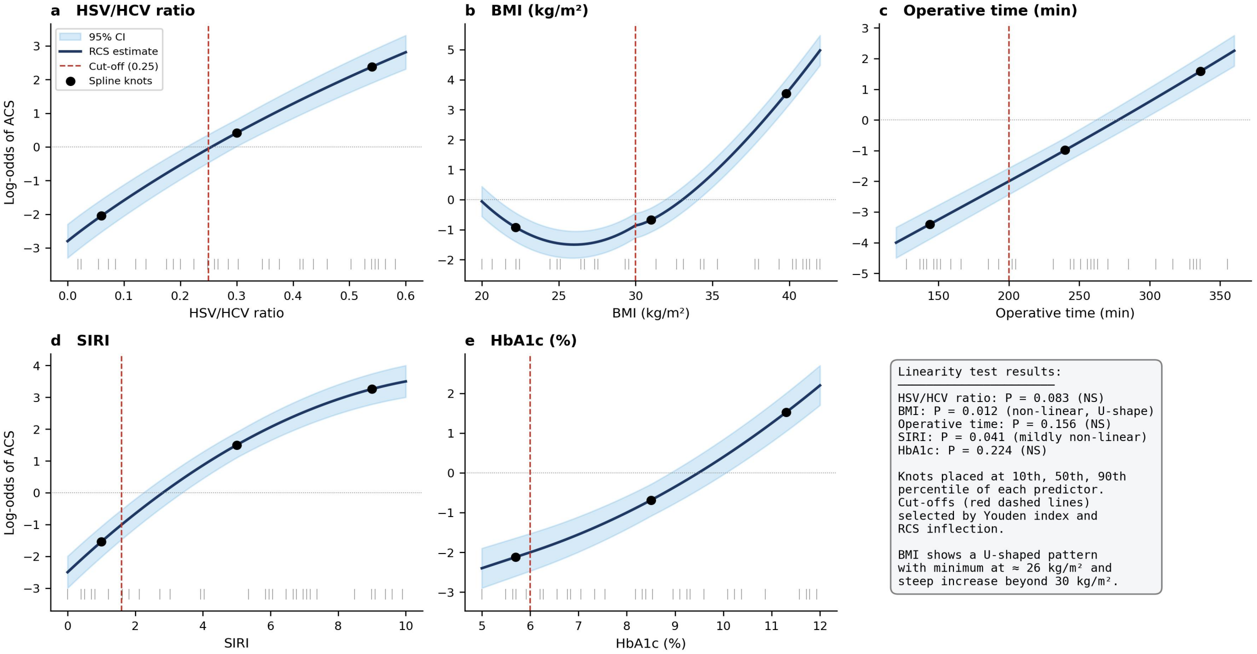

SUPPLEMENTARY FIGURE S1 | Restricted cubic spline (RCS) regression plots showing the relationship between continuous predictors and the log-odds of abdominal compartment syndrome (ACS) in the training cohort (n = 323).

(a) HSV/HCV ratio; (b) BMI; (c) operative time; (d) SIRI; (e) HbA1c. Solid line: RCS estimate; shaded band: 95% CI; red dashed line: selected dichotomization cut-off. Knots (black dots) were placed at the 10th, 50th, and 90th percentile of each predictor. Rug plots at the bottom of each panel show the distribution of observations. Cut-offs were selected by combining published clinical thresholds, RCS inflection points, and Youden-index optimization. The U-shaped BMI relationship (panel b) reflects the well-described "obesity paradox" with minimum risk at approximately 26 kg/m² and steep escalation beyond 30 kg/m². ACS, abdominal compartment syndrome; BMI, body mass index; HbA1c, glycated hemoglobin; HSV/HCV, hernia sac volume to abdominal cavity volume; RCS, restricted cubic spline; SIRI, systemic inflammatory response index.

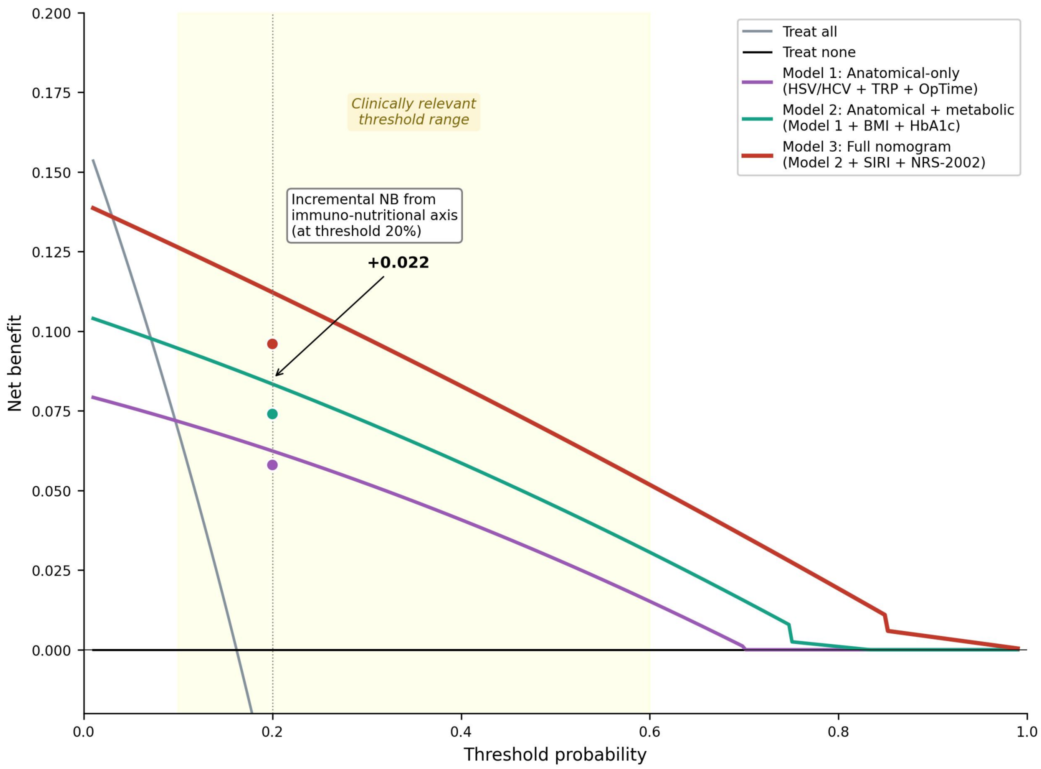

SUPPLEMENTARY FIGURE S2 | Decision curve analysis (DCA) comparing nested models for predicting abdominal compartment syndrome (ACS) across the full study cohort (N = 555).

Three nested models are compared: Model 1 (anatomical-only) includes HSV/HCV ratio, tension reduction procedure (TRP), and operative time (OpTime); Model 2 (anatomical + metabolic) adds BMI and HbA1c; Model 3 (full nomogram) further adds SIRI and NRS-2002. The shaded yellow region indicates the clinically relevant threshold range (10%–60%). At a threshold of 20%, the full nomogram provided a net benefit of 0.096 versus 0.074 for Model 2 and 0.058 for Model 1, corresponding to an incremental net benefit of 0.022 attributable to the immuno-nutritional axis. BMI, body mass index; HbA1c, glycated hemoglobin; HCV, abdominal cavity volume; HSV, hernia sac volume; NB, net benefit; NRS-2002, Nutritional Risk Screening 2002; SIRI, systemic inflammatory response index; TRP, tension reduction procedure.

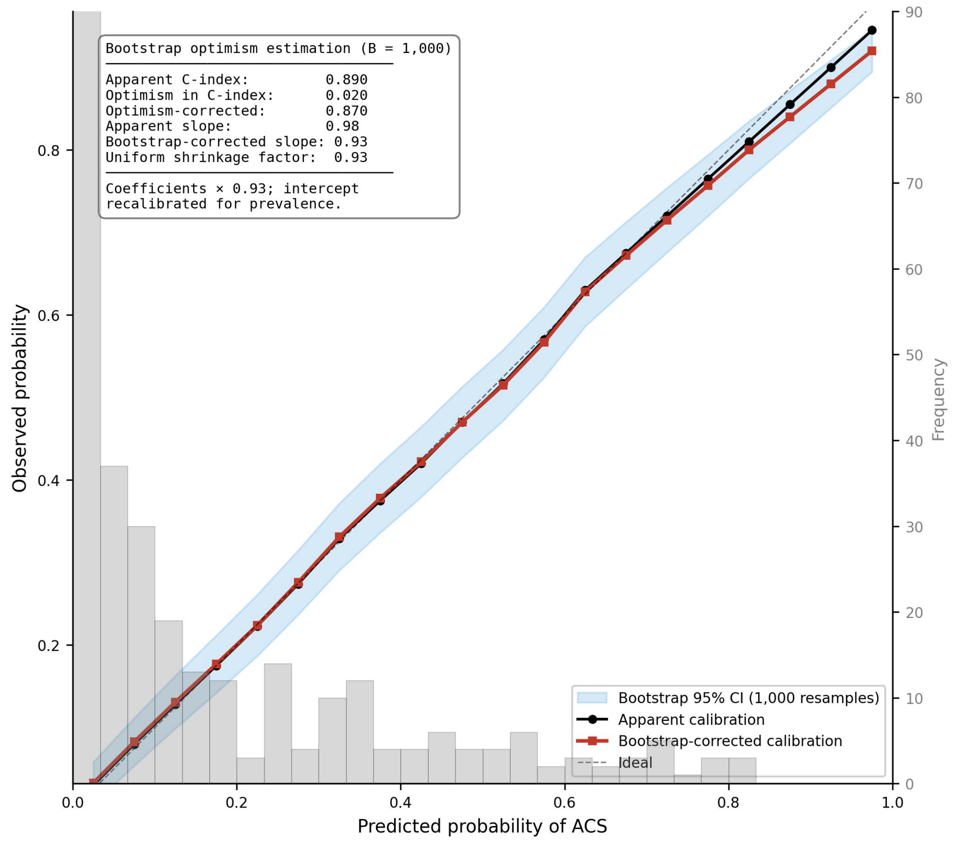

SUPPLEMENTARY FIGURE S3 | Bootstrap-corrected calibration plot of the immuno-nutritional nomogram in the training cohort (n = 323; 53 ACS events).

The blue band shows the 95% confidence interval from 1,000 bootstrap resamples. The black solid line shows the apparent calibration; the red solid line shows the bootstrap-corrected (optimism-adjusted) calibration. The gray dashed line is the ideal diagonal. The histogram at the bottom (gray) shows the distribution of predicted probabilities across the training cohort. Inset: apparent C-index 0.890, optimism 0.020, optimism-corrected C-index 0.870, apparent calibration slope 0.98, bootstrap-corrected slope 0.93, uniform shrinkage factor 0.93. Coefficients of the final model were multiplied by the shrinkage factor and the intercept was recalibrated to the cohort prevalence prior to applying the model to the testing and validation cohorts. CI, confidence interval.

ACS, abdominal compartment syndrome; AIC, Akaike information criterion; AUC, area under the curve; CI, confidence interval; EPV, events per variable; rms, R package for regression modeling strategies.
