# Supplementary material for: Synergistic impact of nutritional risk, glycemic control, and systemic inflammation on Abdominal Compartment Syndrome in diabetic patients following complex ventral hernia repair: a development and validation study
Source: Front Nutr. 2026 Jun 19;13:1786526. doi: 10.3389/fnut.2026.1786526 (PMC13328177; doi:10.3389/fnut.2026.1786526)
Supplement: Supplementary file 2 [file Data_Sheet_2.pdf]

Supplementary Material

Supplementary Tables S1–S5

Table S1 documents the events-per-variable (EPV) calculation and addresses the concern of overfitting (reviewer major comment 2).

Table S2 presents the LASSO penalized regression coefficients alongside the backward-AIC coefficients used in the main analysis (reviewer major comment 2).

Table S3 reports variance inflation factors (VIF) for all final predictors (reviewer major comment 2).

Table S4 compares the dichotomized model with a sensitivity-analysis model in which the same predictors are entered as continuous variables (reviewer major comment 3).

Table S5 presents a sensitivity analysis using alternative HbA1c cut-offs (6.0%, 6.5%, 7.0%) and a continuous parameterization (reviewer major comment 3).

Supplementary Table S1

TABLE S1 | Events-per-variable (EPV) calculation across analysis cohorts.

Cohort	*n*	ACS events, *n* (%)	Candidate predictors at univariate screening	Predictors retained in final model	EPV[a]	Interpretation[b]
Training (model derivation)	323	53 (16.4)	17	7	7.6	Borderline; justifies penalized estimation
Internal testing	138	21 (15.2)	—	7	3.0	Used for testing only; not for estimation
Prospective temporal validation	94	16 (17.0)	—	7	2.3	Used for validation only; not for estimation
**Pooled Phase I derivation**	**461**	**74 (16.1)**	**17**	**7**	**10.6**	**Meets Peduzzi's 10-EPV threshold**

[a] EPV = number of ACS events / number of predictors retained in the final multivariable model (7).

[b] Per Peduzzi et al. (1996), an EPV ≥ 10 is the conventional minimum to limit overfitting in multivariable logistic regression. The training cohort EPV of 7.6 is borderline; we therefore performed LASSO penalized regression with bootstrap optimism correction as a pre-specified sensitivity analysis (Table S2).

ACS, abdominal compartment syndrome; EPV, events-per-variable.

Supplementary Table S2

TABLE S2 | Comparison of regression coefficients between backward-AIC selection (main analysis) and LASSO penalized regression (sensitivity analysis).

Predictor	AIC β (SE)	AIC OR (95% CI)	AIC *P*	LASSO β (1-SE λ)[a]	LASSO OR	Shrinkage[b]
Final model coefficients (training cohort, n = 323)						
Intercept (β_0)	−4.350 (0.624)	—	—	−4.180	—	3.9%
HSV/HCV ratio ≥ 0.25	1.012 (0.288)	2.75 (1.60–4.85)	<0.001	0.870	2.39	14.0%
Tension reduction procedure	0.896 (0.298)	2.45 (1.38–4.42)	0.003	0.780	2.18	12.9%
Operative time > 200 min	0.751 (0.286)	2.12 (1.20–3.70)	0.010	0.631	1.88	16.0%
BMI ≥ 30 kg/m²	0.631 (0.267)	1.88 (1.12–3.25)	0.018	0.519	1.68	17.7%
NRS-2002 ≥ 3	0.779 (0.270)	2.18 (1.30–3.70)	0.004	0.658	1.93	15.5%
SIRI ≥ 1.6	0.683 (0.269)	1.98 (1.18–3.45)	0.011	0.583	1.79	14.6%
HbA1c ≥ 6.0%	0.501 (0.241)	1.65 (1.03–2.64)	0.038	0.412	1.51	17.8%
Apparent model performance (training cohort)						
AUC-ROC	0.890	—	—	0.881	—	—
Brier score	0.092	—	—	0.094	—	—
Calibration slope	0.98	—	—	1.00 (by design)	—	—
Number of non-zero predictors	7	—	—	7	—	—
Prospective temporal validation cohort (n = 94)						
AUC-ROC (95% CI)	0.84 (0.77–0.91)	—	—	0.83 (0.76–0.90)	—	—
Brier score	0.112	—	—	0.114	—	—

[a] LASSO logistic regression was performed using the glmnet R package with 10-fold cross-validation. The penalty parameter λ was selected by the 1-SE rule ($\lambda_{1\text{-SE}}$ = 0.0382). At this λ, all seven predictors retained non-zero coefficients, identical to the variables retained by backward AIC selection.

[b] Absolute shrinkage = 1 − (LASSO β / AIC β); reported as percentage. Shrinkage of 12–18% indicates that the AIC coefficients are mildly optimistic but not the product of unstable variable selection.

AIC, Akaike information criterion; AUC-ROC, area under the receiver operating characteristic curve; BMI, body mass index; CI, confidence interval; HbA1c, glycated hemoglobin; HCV, abdominal cavity volume; HSV, hernia sac volume; LASSO, least absolute shrinkage and selection operator; NRS-2002, Nutritional Risk Screening 2002; OR, odds ratio; SE, standard error; SIRI, systemic inflammatory response index.

Supplementary Table S3

TABLE S3 | Variance inflation factor (VIF) and tolerance for the seven final predictors in the training cohort.

Predictor	Tolerance (1 / VIF)	VIF	Interpretation[a]
HbA1c ≥ 6.0%	0.92	1.09	Negligible
HSV/HCV ratio ≥ 0.25	0.85	1.18	Negligible
BMI ≥ 30 kg/m²	0.82	1.22	Negligible
Operative time > 200 min	0.76	1.31	Negligible
Tension reduction procedure	0.70	1.42	Mild
SIRI ≥ 1.6	0.68	1.48	Mild
NRS-2002 ≥ 3	0.65	1.55	Mild
**Mean VIF**	—	**1.32**	**Below the 5.0 cut-off**

[a] Interpretation conventions: VIF < 2, negligible collinearity; VIF 2.0–5.0, mild collinearity; VIF > 5.0, problematic collinearity warranting variable removal or combination. All values were below 2.0 and therefore well below the conventional 5.0 cut-off.

BMI, body mass index; HbA1c, glycated hemoglobin; HCV, abdominal cavity volume; HSV, hernia sac volume; NRS-2002, Nutritional Risk Screening 2002; SIRI, systemic inflammatory response index; VIF, variance inflation factor.

Supplementary Table S4

TABLE S4 | Sensitivity analysis: comparison of model performance and effect estimates between a continuous-variable model and the dichotomized model used in the main analysis.

**Metric / Predictor**	**Continuous-variable model[a]**	**Dichotomized model (main analysis)**
A. Model performance		
AUC-ROC, training (95% CI)	0.88 (0.84–0.93)	0.89 (0.85–0.93)
AUC-ROC, internal testing (95% CI)	0.85 (0.79–0.92)	0.86 (0.80–0.92)
AUC-ROC, prospective temporal validation (95% CI)	0.83 (0.76–0.90)	0.84 (0.77–0.91)
Brier score, training	0.094	0.092
Calibration slope, training (95% CI)	0.97 (0.84–1.10)	0.98 (0.85–1.11)
Hosmer–Lemeshow *P*, training	0.71	0.85
B. Coefficient comparison (training cohort, multivariable logistic regression)		
HSV/HCV ratio	OR 1.54 (1.31–1.81) per 0.10 increase	OR 2.75 (1.60–4.85) for ≥ 0.25
BMI	OR 1.087 (1.024–1.154) per 1 kg/m²	OR 1.88 (1.12–3.25) for ≥ 30 kg/m²
Operative time	OR 1.31 (1.13–1.53) per 30 min	OR 2.12 (1.20–3.70) for > 200 min
SIRI	OR 1.23 (1.09–1.40) per 1 unit	OR 1.98 (1.18–3.45) for ≥ 1.6
HbA1c	OR 1.20 (1.04–1.39) per 1%	OR 1.65 (1.03–2.64) for ≥ 6.0%

[a] The continuous-variable model used the same seven candidate predictors as the main model, with HSV/HCV ratio, BMI, operative time, SIRI, and HbA1c entered as continuous variables; tension reduction procedure (binary) and NRS-2002 ≥ 3 (binary, per ESPEN definition) remained unchanged. Restricted cubic splines (3 knots at the 10th, 50th, and 90th percentiles) confirmed monotonic, near-linear relationships for all five continuous predictors after appropriate transformation (Supplementary Figure S1). The discrimination of the continuous-variable model was within 0.01 AUC unit of the dichotomized model in all cohorts, supporting the use of dichotomized cut-offs in the nomogram for clinical applicability.

AUC-ROC, area under the receiver operating characteristic curve; BMI, body mass index; CI, confidence interval; ESPEN, European Society for Clinical Nutrition and Metabolism; HbA1c, glycated hemoglobin; HCV, abdominal cavity volume; HSV, hernia sac volume; NRS-2002, Nutritional Risk Screening 2002; OR, odds ratio; SIRI, systemic inflammatory response index.

Supplementary Table S5

TABLE S5 | Sensitivity analysis using alternative HbA1c thresholds for the multivariable model.

HbA1c parameterization	Patients above threshold, *n* (%)	Adjusted OR (95% CI)	*P*	Other 6 predictors retained[a]	Training AUC	Validation AUC
**≥ 6.0% (main analysis)**	**189 (41.0)**	**1.65 (1.03–2.64)**	**0.038**	**All 6 retained**	**0.890**	**0.840**
≥ 6.5%	156 (33.8)	1.78 (1.10–2.88)	0.019	All 6 retained	0.890	0.842
≥ 7.0%	124 (26.9)	1.92 (1.16–3.18)	0.011	All 6 retained	0.884	0.831
Continuous (per 1% increase)	—	1.20 (1.04–1.39)	0.013	All 6 retained	0.881	0.834

[a] The other six predictors entered into each sensitivity model were identical to those in the main analysis (HSV/HCV ratio ≥ 0.25, tension reduction procedure, operative time > 200 min, BMI ≥ 30 kg/m², NRS-2002 ≥ 3, SIRI ≥ 1.6). "All 6 retained" indicates that all six predictors maintained $P < 0.05$ in the multivariable model under each HbA1c parameterization. The chosen 6.0% threshold for the main analysis reflects the American Diabetes Association diagnostic cut-off and provides clinically actionable guidance for perioperative glycemic optimization. Higher thresholds yielded numerically larger odds ratios for HbA1c but did not meaningfully change discrimination (ΔAUC ≤ 0.01) or alter the qualitative ranking of predictor importance.

AUC, area under the curve; BMI, body mass index; CI, confidence interval; HbA1c, glycated hemoglobin; HCV, abdominal cavity volume; HSV, hernia sac volume; NRS-2002, Nutritional Risk Screening 2002; OR, odds ratio; SIRI, systemic inflammatory response index.
